# A host defense role for Fibrinogen by direct binding of *Clostridium botulinum* C2 toxin

**DOI:** 10.1007/s00018-026-06253-7

**Published:** 2026-05-19

**Authors:** Sophia Kistermann, Sebastian Heber, Stephan Fischer, Jannik Sichau, Christoph Q. Schmidt, Marco Mannes, Markus Huber-Lang, Holger Barth

**Affiliations:** 1 Institute of Experimental and Clinical Pharmacology, Toxicology and Pharmacology of Natural Products, Ulm University Medical Center, 89081 Ulm, Germany; 2 Institute of Clinical and Experimental Trauma Immunology, Ulm University Medical Center, 89081 Ulm, Germany

**Keywords:** Bacterial protein toxin, Blood coagulation factor, C2 toxin, *Clostridium botulinum*, Fibrinogen, Toxin inhibitor

## Abstract

In addition to fibrinogens canonical function in hemostasis, alternate roles in innate immunity have emerged. However, interactions with bacterial protein toxins, the main virulence factors of many medically relevant bacteria and causative agents of life-threatening diseases, have not been described so far. Here, we identified human fibrinogen as an inhibitor of the enterotoxic *Clostridium* (*C.*) *botulinum* C2 toxin. Our results indicate that fibrinogen specifically interacts with the binding subunit of C2 toxin in vitro and that N-linked glycans of fibrinogen are crucial for this interaction. This prevents receptor-binding and cellular uptake of the toxin. Related toxins from *Bacillus anthracis* (lethal toxin), *C. perfringens* (iota) or *Clostridioides difficile* (CDT) do not bind to fibrinogen and are therefore not inhibited. Furthermore, C2 toxin had no effect on coagulation of human blood ex vivo. In conclusion, we identified fibrinogen as an inhibitor of a highly potent bacterial protein toxin, highlighting an unexpected role for this blood coagulation factor in innate immunity.

**Supplementary Information:**

The online version contains supplementary material available at 10.1007/s00018-026-06253-7.

## Introduction

Bacterial AB-type exotoxins are highly toxic proteins and therefore serve as important virulence factors of bacteria due to their unique structure and mode of action. They contain functionally different subunits. The binding and translocation (B) subunit mediates binding to human or animal cells and triggers subsequent internalization, while the active (A) subunit harbors enzyme activity. After their secretion from the producing bacteria, AB-toxins enter their target cells via receptor-mediated endocytosis and deliver their A-subunit into the cytosol. The enzymatic reaction catalyzed by the A-subunit leads to structural and/or functional changes of the individual substrate molecules, which alters cell functions, resulting in the characteristic clinical symptoms caused by the individual toxin [1]. AB-type toxins are responsible for many life-threatening human and animal diseases including diphtheria, cholera, pertussis, anthrax, tetanus, botulism and enterocolitis. Adequate clinical treatment, which includes antibiotics and supportive care, is often limited to manage these diseases and therefore, novel therapeutic options are urgently needed including compounds to specifically and directly neutralize the toxins that cause the disease [2–4].

The C2 enterotoxin is the prototype of binary toxins. It is produced by *Clostridium (C.) botulinum*, the pathogen responsible for botulism [1, 5]. C2 toxin consists of two individual proteins, C2I and C2II, which assemble in solution or on the surface of target cells to form biologically active C2 toxin [6]. C2I is the A-subunit that ADP-ribosylates G-actin [7, 8]. C2II is the B-subunit that requires proteolytic activation to form the biologically active C2IIa, which assembles to homo-heptamers [9, 10]. C2 toxin binds via C2IIa to cell surface receptors, which are complex and hybrid asparagine-linked carbohydrate structures present on all mammalian cell types [11]. This triggers receptor-mediated endocytosis and C2 toxin is internalized into early endosomal vesicles [9, 12]. Upon endosomal acidification, C2IIa changes its conformation and inserts as a trans-membrane pore into endosomal membranes, which facilitates the transport of C2I into the cytosol [9, 13, 14]. There, C2I mono-ADP-ribosylates G-actin, which inhibits actin polymerization and disrupts actin-mediated cell functions [7, 15]. C2 toxin-treated cells round up, cell-cell contacts and epithelial barriers are disrupted, and finally, cells undergo apoptosis [16–21]. Due to this mode of action, C2 toxin and the other binary actin ADP-ribosylating toxins, *Clostridioides difficile* CDT and *C. perfringens* iota toxin, act as enterotoxins in humans and animals. Although C2 toxin is not the causative agent of human botulism, C2 toxin producing *C. botulinum* strains C and D play a role in animal outbreaks of botulism and hold the potential to spread to humans [22, 23].

There is increasing evidence, also from our group, that the human innate immune system might be able to directly fight bacterial exotoxins through body-own proteins and peptides, which were in particular found in the blood. It was demonstrated that serum albumin, specific defensins and α-1-antitrypsin neutralize medically relevant exotoxins including diphtheria toxin, pertussis toxin, anthrax toxin, and the *Clostridioides difficile* toxins TcdA, TcdB and CDT in cell models, human organoids and animals [24–36]. Most of these proteins or peptides directly and specifically bind to the toxins, preventing key functions such as toxin binding to cells or enzymatic activity, thereby protecting cells from intoxication. As human body-own proteins, these toxin-inhibitors should lack toxic or immunological issues after their application to patients. Therefore, and in front of the increasing issues with bacterial resistance against established anti-bacterial drugs [37], they should represent highly attractive candidates to develop novel therapeutic options to treat or prevent toxin-associated diseases. Since there is intensive crosstalk between the coagulation and the innate immune defense system [38, 39], it is tempting to speculate that central components of the coagulation system may not only activate cellular and fluid phase immunity and interact with bacteria but also directly interact with defined toxins.

Here, we report for the first time that a human blood coagulation factor serves as a potent and specific inhibitor of a bacterial protein toxin. We discovered that fibrinogen (Fib) specifically neutralizes the cytotoxic activity of *C. botulinum* C2 toxin and performed an in-depth analysis of the molecular mechanisms underlying this toxin-inhibiting effect of Fib.

## Results

### Fib protects HeLa cells from intoxication with C2 toxin

Blood coagulation factors, as one major class of proteins in the human blood, were not described in the context of toxin-neutralizing molecules in innate immunity. We and others previously identified other proteins from human blood such as albumin as inhibitors of bacterial protein toxins [26]. Hence, we tested Fib, the most abundant coagulation factor in human blood, for its activity against bacterial AB-type exotoxins.

**Fig. 1:**
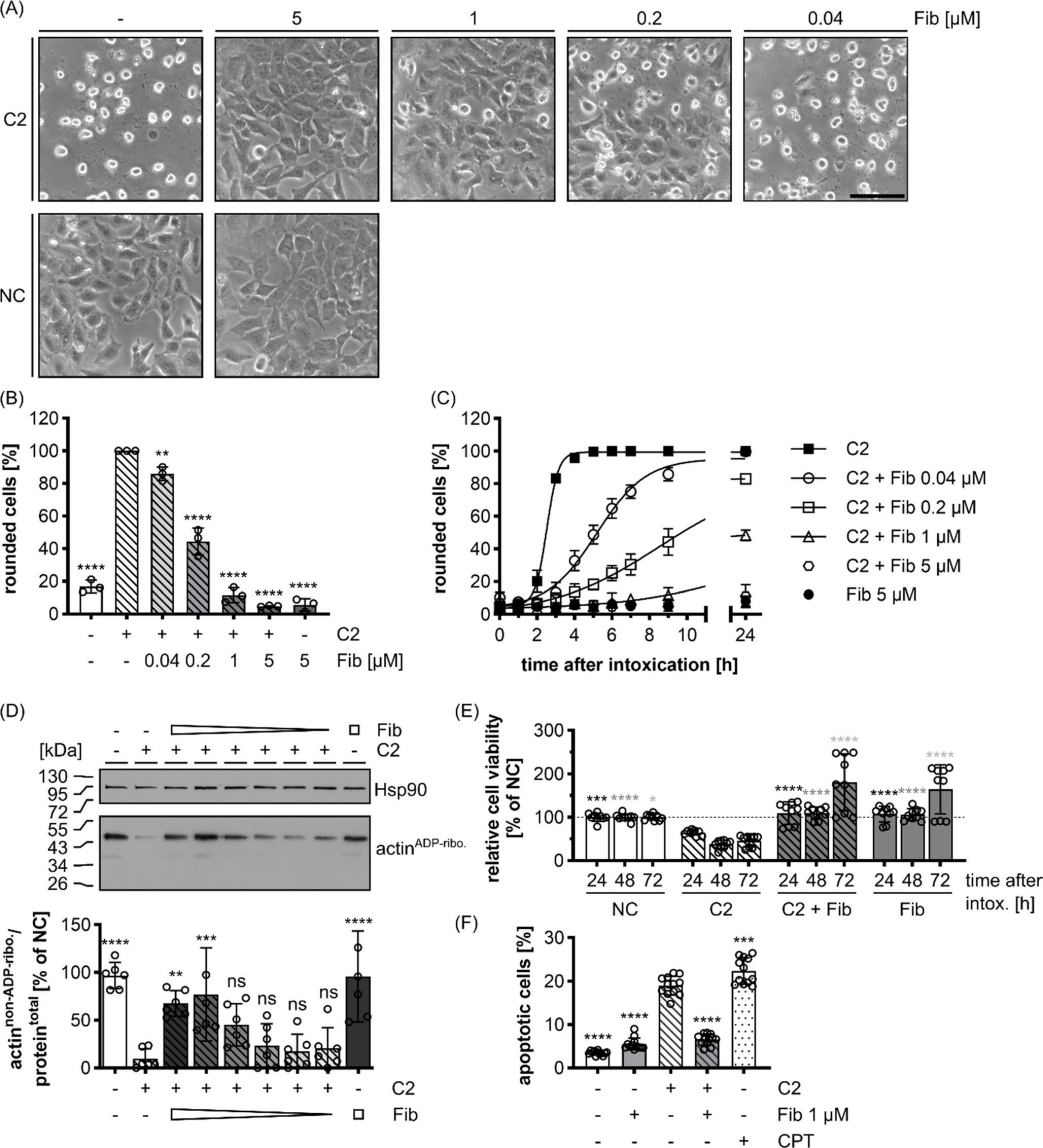
Fib protects HeLa cells from intoxication with C2 toxin. (**A**) Morphological analysis: representative pictures of HeLa cells after 9 h treatment with C2 toxin (2 nM C2I + 3.32 nM C2IIa) in the absence or presence of Fib (concentrations are indicated). Untreated cells served as a negative control (NC). Scale bar corresponds to 100 μm. (**B**) Quantification of the results from (A). (**C**) Quantification of the C2 toxin-induced cell-rounding in the absence or presence of Fib in relation to the time after intoxication. Quantifications from the same experiment as (A). Pictures were taken at the indicated time points and the percentage of rounded cells from the total cell number was quantified. Values are given as mean ± SD (*n* = 3) of triplicates from one representative experiment. (**D**) Upper panel: biochemical analysis of the effect of Fib on intoxication of cells with C2 toxin. Western blot of the actin ADP-ribosylation status (actin^ADP-ribo^.) of HeLa cells after treatment with C2 toxin in the absence or presence of Fib. HeLa cells were incubated with Fib (1000 nM, 200 nM, 40 nM, 8 nM, 1.6 nM, 0.32 nM, ) and C2 toxin (2 nM C2I + 3.32 nM C2IIa) as indicated for 24 h. Cells were harvested and the actin ADP-ribosylation status was probed. Hsp90 was used to show equal loading. Notably, a weak actin signal indicates a strong C2 toxin activity in intact cells. Lower panel: quantification of all Western blots. The amount of ADP-ribosylated actin was normalized to total protein amount. Three individual experiments with duplicates were quantified (*n* = 6). (**E**) Cells were treated as indicated with Fib (5 µM), C2 toxin (2 nM C2I + 3.32 nM C2IIa) or left untreated for negative control (NC). Cell viability was measured by using the MTS assay for each condition after one, two and three days and normalized for every timepoint to the NC. Values are given as mean ± SD (*n* = 9) of triplicates from three individual experiments. Statistical analysis was always performed within the respective timepoints (depicted as differently shaded significance symbols in black, dark grey and light grey for 24 h, 48 h and 72 h respectively). (**F**) Quantification of the percentage of apoptotic cells. Cells were treated for 24 h with Fib (1 µM), C2 toxin (2 nM C2I + 3.32 nM C2IIa) or camptothecin (CPT, 10 µM) as indicated. Cells were detached, stained and analyzed in the flow cytometer for apoptosis (Annexin-V positive, Sytox negative). The percentage of apoptotic cells are given as mean ± SD (*n* = 12) of triplicates from four individual experiments. Statistical analysis was performed compared to the C2 control by using non-parametric one-way ANOVA in combination with Dunnett’s correction for multiple comparison (ns *p* ≥ 0.05, * *p* < 0.05, ** *p* < 0.01, *** *p* < 0.001, **** *p* < 0.0001)

In a first set of experiments, the effect of Fib on the intoxication of HeLa cells with C2 toxin was analyzed. Fib concentrations below 5 µM were chosen as a starting point, which are equivalent or below Fib plasma levels found in healthy individuals (ranging from 200 to 400 mg/dl or 5.9 to 11.8 µM [40, 41]). C2 cytotoxicity manifests in cultured cells as cell rounding that serves as highly sensitive and specific endpoint for the intoxication and can easily be quantified. When C2 toxin was applied in combination with Fib, cytotoxicity was significantly reduced in a time- and concentration-dependent manner as shown by a lower number of rounded cells compared to cells treated with C2 toxin in the absence of Fib (Fig. 1A-C). Fib concentrations comparable to the human plasma level prevented C2 toxin-mediated cytotoxicity over 24 h. To show that this effect was associated with the modification of actin by C2 toxin in the cells, the ADP-ribosylation status of actin was assessed after 24 h (Fig. 1D). Therefore, the treated cells were lysed and subjected to a post ADP-ribosylation reaction with biotin-labeled NAD^+^, in which the cellular actin that was not ADP-ribosylated during the incubation of the living cells with C2 toxin, became ADP-ribosylated and therefore biotin-labeled in vitro. Hence, a strong actin^ADP−ribo^. signal corresponds to low toxicity of C2 toxin in living cells. The protective effects seen before by cell morphology analysis were confirmed by this biochemical approach, as a significantly reduced ADP-ribosylation of actin by C2 toxin in the living cells was observed when cells were incubated with C2 toxin in the presence of Fib. Taken together, these results indicate that the presence of Fib in the medium prevented the intoxication of cells by C2 toxin, as demonstrated by both morphological and biochemical analysis. C2 toxin does not kill cells immediately, but the toxin-induced depolymerization of the actin cytoskeleton and associated inhibition of actin-dependent processes, results in delayed cell death [17]. Since Fib protected HeLa cells efficiently from the early cytotoxic effects of C2 toxin, namely actin ADP-ribosylation and cell rounding, its influence on the delayed C2 toxin-induced cell death was investigated. Over all observed timepoints, 5 µM Fib completely prevented the C2 toxin-induced cell viability loss (Fig. 1E, Supplementary Fig. 1A). Fib alone had no effect on cell viability and even increased the amount of viable cells at the latest timepoint. This effect was observed even if C2 toxin was present. The protective effects of Fib were also observed for apoptotic cell death upon C2 toxin treatment after 24 h (Fig. 1F, Supplementary Fig. 1B). In this approach, camptothecin (CPT) was used as a positive control, which is known to induce apoptosis in HeLa cells [42]. A highly significant decrease in apoptotic cells was measured when C2 toxin was concomitantly applied with Fib, confirming that Fib protects cells from C2 toxin.

### Fib protects CaCo-2 cells from intoxication with C2 toxin

**Fig. 2:**
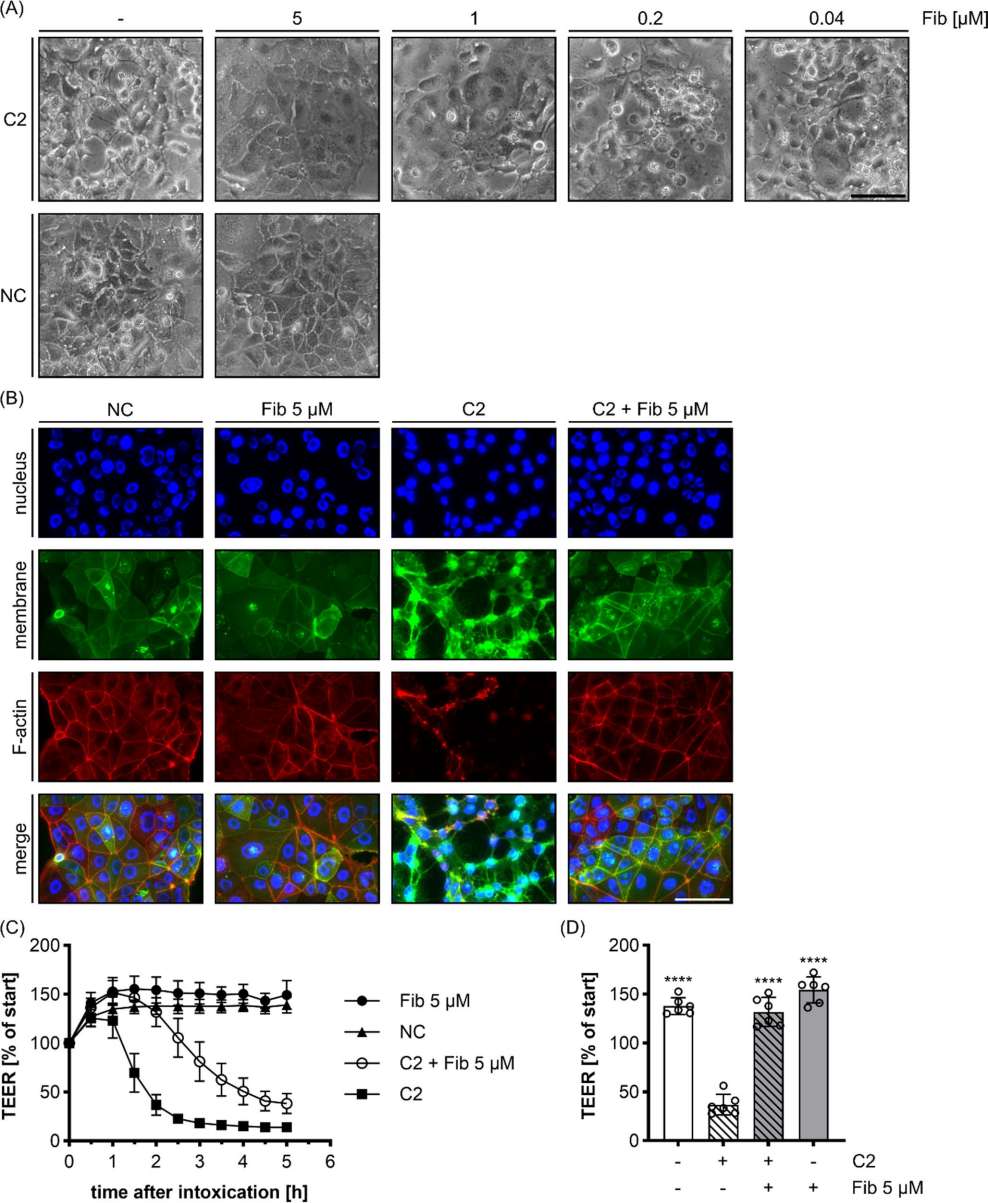
Fib protects CaCo-2 cells from intoxication with C2 toxin and ameliorates C2 toxin-induced epithelial barrier dysfunction. (**A**) Morphological analysis of the effect of Fib on the intoxication of CaCo-2 cells with C2 toxin. Shown are representative pictures of CaCo-2 cells after 7 h incubation with C2 toxin (4 nM C2I + 6.64 nM C2IIa) in the absence or presence of increasing Fib concentrations as indicated. Untreated cells served as a negative control (NC). Scale bar corresponds to 100 μm. (**B**) Representative pictures from fluorescence microscopic analysis of CaCo-2 cells treated with C2 toxin (8 nM C2I + 13.28 nM C2IIa) in the absence or presence of Fib (5 µM). After 5 h, cells were fixed and nucleus, membrane and F-actin were specifically stained for fluorescence microscopy. Scale bar corresponds to 100 μm. (**C**) Transepithelial electrical resistance (TEER) of an untreated CaCo-2 cell monolayer, and after its treatment with C2 toxin (1 nM C2I + 1.66 nM C2IIa) in the absence or presence of Fib (5 µM). For control, cells were incubated with Fib (5 µM) alone or left untreated (NC). The TEER of each condition was measured as indicated and normalized to the respective TEER value of each condition at 0. Values are given as mean ± SD (*n* = 6) of duplicates from three individual experiments. (**D**) Bar graph of the values at the 2 h timepoint from (C). Statistical analysis was performed compared to C2 toxin-treated cells by using non-parametric one-way ANOVA in combination with Dunnett’s correction for multiple comparison (**** *p* < 0.0001)

Prompted by these findings, the effect of Fib on C2 toxin was investigated in the pathophysiological more relevant human intestine-derived cell line CaCo-2. As observed for HeLa cells, Fib prevented the intoxication of these cells with C2 toxin in a concentration dependent manner, as analyzed by morphology changes of CaCo-2 cells (Fig. 2A). To better visualize the effect of C2 toxin and the protective effect of Fib against C2 toxin on the integrity of the tight epithelial monolayer formed by the CaCo-2 cells, immunofluorescence microscopy was performed with nucleus, membrane and F-actin staining (Fig. 2B). Confluently grown CaCo-2 cells build an epithelial monolayer with tight cell contacts in between the individual cells, which serves as well-established model for the investigation of epithelial barrier functions. These cell contacts are closely associated to their accompanying cortical actin network that is most strongly stained at the cell-cell borders. Upon C2 toxin treatment, F-actin depolymerizes and is no longer detectable as a homogeneous cortex in the cell. As a consequence, cells lose their characteristic morphology, detach from each other and gaps are formed between the cells. However, the membranes of cells intoxicated by C2 toxin were still detectable. The C2 toxin-induced loss of barrier integrity due to C2 toxin treatment was further demonstrated by the measurement of transepithelial electrical resistance (TEER), a highly sensitive and quantitative endpoint (Fig. 2C). After C2 toxin-treatment, the epithelial monolayer lost TEER as soon as 1 h after treatment and reached minimal values after 3 h. However, in the presence of Fib, the decrease of TEER due to C2 toxin was significantly delayed, in comparison to C2 alone (Fig. 2D), clearly indicating a protective effect of Fib towards C2 toxin-induced cell rounding and loss of epithelial barrier function even in this highly sensitive approach.

### Fib does not inhibit closely related binary AB-type toxins

**Fig. 3:**
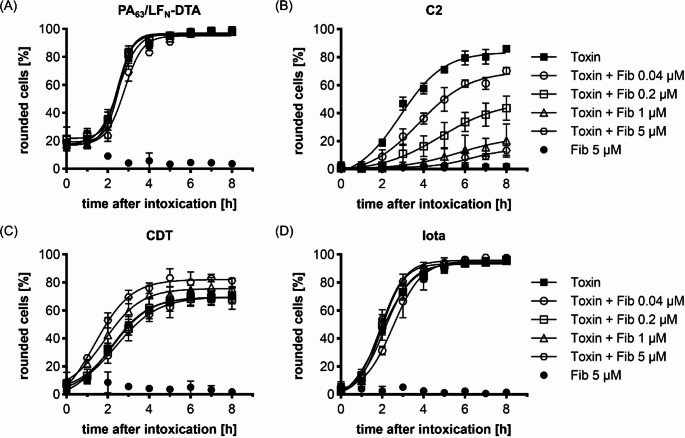
Fib does not inhibit the closely related toxins PA_63_/LF_N_-DTA, CDT or Iota. Quantification of cytotoxicity assay of cells treated with (**A**) PA_63_/LF_N_-DTA (0.5 nM PA_63_ + 0.25 nM LF_N_-DTA), (**B**) C2 toxin (2 nM C2I + 3.32 nM C2IIa), (**C**) CDT (2.8 nM CDTa + 3 nM CDTb) or (**D**) Iota (0.7 nM Ia + 0.94 nM Ib). Cells (HeLa for (A), Vero for (B), (C) and (D)) were treated with the respective toxin in the absence or presence of the indicated concentration of Fib. Untreated cells served as a negative control (NC). Pictures were taken at the indicated time points and the percentage of rounded cells from the total cell number was quantified. Values are given as mean ± SD (*n* = 3) of triplicates from one representative experiment

Intrigued by its robust protective effect against C2 toxin, we investigated whether Fib also inhibits other related members of the family of binary AB-type toxins. The closest related member to C2IIa is the protective antigen (PA_63_) from *Bacillus anthracis* anthrax toxin [43]. We investigated PA_63_ dependent cytotoxicity by combining PA_63_ with the well-established chimeric toxin LF_N_-DTA. LF_N_-DTA combines the N-terminus from the natural A-component lethal factor (LF_N_) that serves as mediator for uptake by PA_63_ and the cytotoxic enzyme domain from diphtheria toxin (DTA) allowing for fast and easy readout on HeLa cells, which round up due to the DTA activity in their cytosol [44–46]. However, as shown in Fig. 3A, Fib had no effect on the intoxication of HeLa cells by PA_63_/LF_N_-DTA, at least not in concentrations that efficiently inhibited C2 toxin. Next, the effect of Fib on *C. difficile* binary toxin (CDT) and *C. perfringens* iota toxin (iota) was tested. These toxins are highly similar to one another and similar to C2 toxin. Like C2I, their enzyme components CDTa and Ia, respectively mono-ADP-ribosylate actin at arginine-177, which results in cell rounding [47–49]. Moreover, their B-subunits CDTb and Ib have similar heptameric structures as C2IIa and share a comparable mode-of-action with C2IIa [50]. CDT and iota use a different cellular receptor for cell entry, namely the lipolysis-stimulated lipoprotein receptor (LSR) [51], while C2 toxin uses a carbohydrate structure [11]. Because LSR is not expressed in HeLa cells, we used Vero cells as a suitable cell line to investigate the effect of Fib on CDT and iota [51]. Before investigating CDT and iota, we verified that C2 toxin was also inhibited by Fib in a concentration and time dependent manner in Vero cells (Fig. 3B). However, Fib had no effect on the intoxication of Vero cells with CDT or iota (Fig. 3C, D). Cell morphological images of selected timepoints for each toxin are depicted in Supplementary Fig. 2. Taken together, Fib robustly neutralizes the cytotoxic activity of C2 toxin, but it has no effect on the related binary toxins, implicating a highly specific inhibitory mode of action towards C2 toxin.

### Fib has no effect on *C. difficile* toxin B (TcdB), but inhibits C2IIa-mediated delivery of its isolated enzyme domain (H_GTD) into the cytosol

**Fig. 4:**
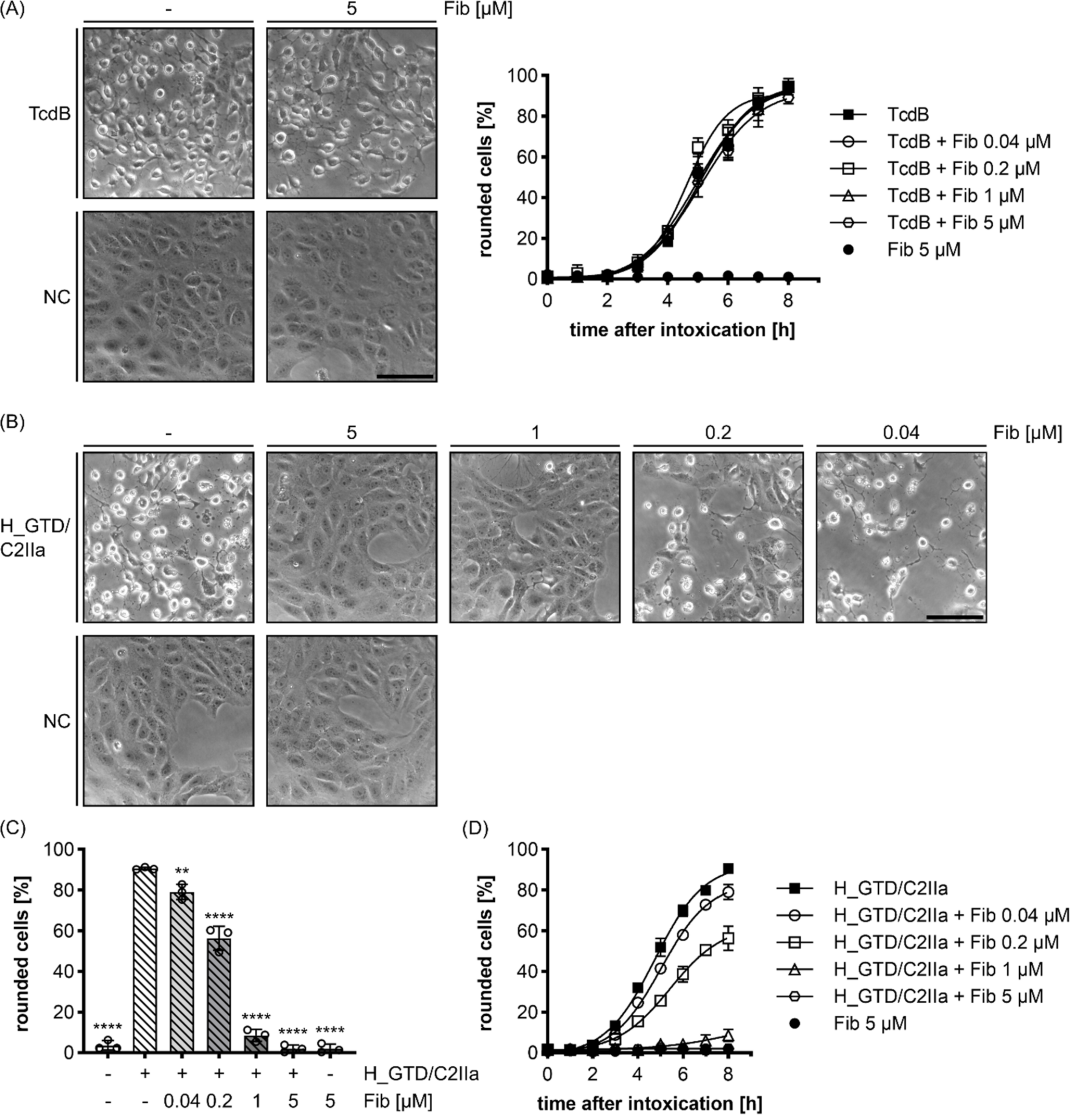
Fib does not inhibit TcdB cytotoxicity on Vero cells but protects from the C2IIa-mediated transport of the glycosyltransferase domain from TcdB (H_GTD). (**A**) Left panel: representative pictures of Vero cells after 8 h treatment with the indicated concentrations of Fib and TcdB (10 pM). Untreated cells served as a negative control (NC). Right panel: pictures were taken at the indicated time points and the percentage of rounded cells from the total cell number was quantified. Values are given as mean ± SD (*n* = 3) of triplicates from one representative experiment. (**B**) Vero cells were treated with indicated concentrations of Fib and H_GTD/C2IIa (200 nM H_GTD + 20 nM C2IIa). Representative pictures after 6 h incubation are shown. (**C**) Quantification of the results from (B). (**D**) Quantification of the H_GTD/C2IIa -induced cell-rounding in the absence or presence of Fib in relation to the time after intoxication. Quantifications from the same experiment as (B). Pictures were taken at the indicated time points and the percentage of rounded cells from the total cell number was quantified. Scale bar corresponds to 100 μm. Statistical analysis was performed compared to the C2 control by using non-parametric one-way ANOVA in combination with Dunnett’s correction for multiple comparison (** *p* < 0.01, **** *p* < 0.0001)

To obtain more detailed information on how Fib inhibits C2 toxin, we included another toxin into this study, namely *C. difficile* TcdB, a single chain protein toxin that glucosylates Rho-GTPases in the cytosol, thereby inhibiting Rho-signaling in cells. As Rho-GTPases are regulators of the actin cytoskeleton and intracellular actin dynamics, TcdB treatment of adherent cells results in cell rounding, which is used as an endpoint to monitor TcdB cytotoxicity [52]. The intoxication of Vero cells with TcdB was not affected by Fib (Fig. 4A), indicating that Fib does not interfere with TcdB cytotoxicity. We demonstrated earlier that C2IIa can serve as a transporter of His-tagged proteins, including the isolated His-tagged glucosyltransferase domain of TcdB (H_GTD), into the cytosol of cells, which then round up as response to the GTD activity in their cytosol [44]. Here, we took advantage of this approach to elucidate the role of C2IIa for the inhibitory interaction with Fib. To this end, Vero cells were incubated with H_GTD plus C2IIa in the absence and presence of increasing concentrations of Fib and the percentage of round cells were determined after different incubation periods. As observed before for C2 toxin, cells were protected against H_GTD/C2IIa by Fib in a concentration- and time-dependent manner (Fig. 4B-D). Fib also inhibited C2IIa-mediated cytotoxicity of the glucosyltransferase domain after 8 h in treatments with H_GTD/C2IIa at concentrations as low as 40 nM (Fig. 4C), which is comparable to C2 toxin. Taken together, these results indicate that Fib did not inhibit TcdB but the delivery of its enzyme domain by C2IIa, implicating that Fib specifically interferes with C2IIa functions.

### Fib does not affect the enzyme activity of C2I but inhibits the binding of C2 toxin to cell

**Fig. 5:**
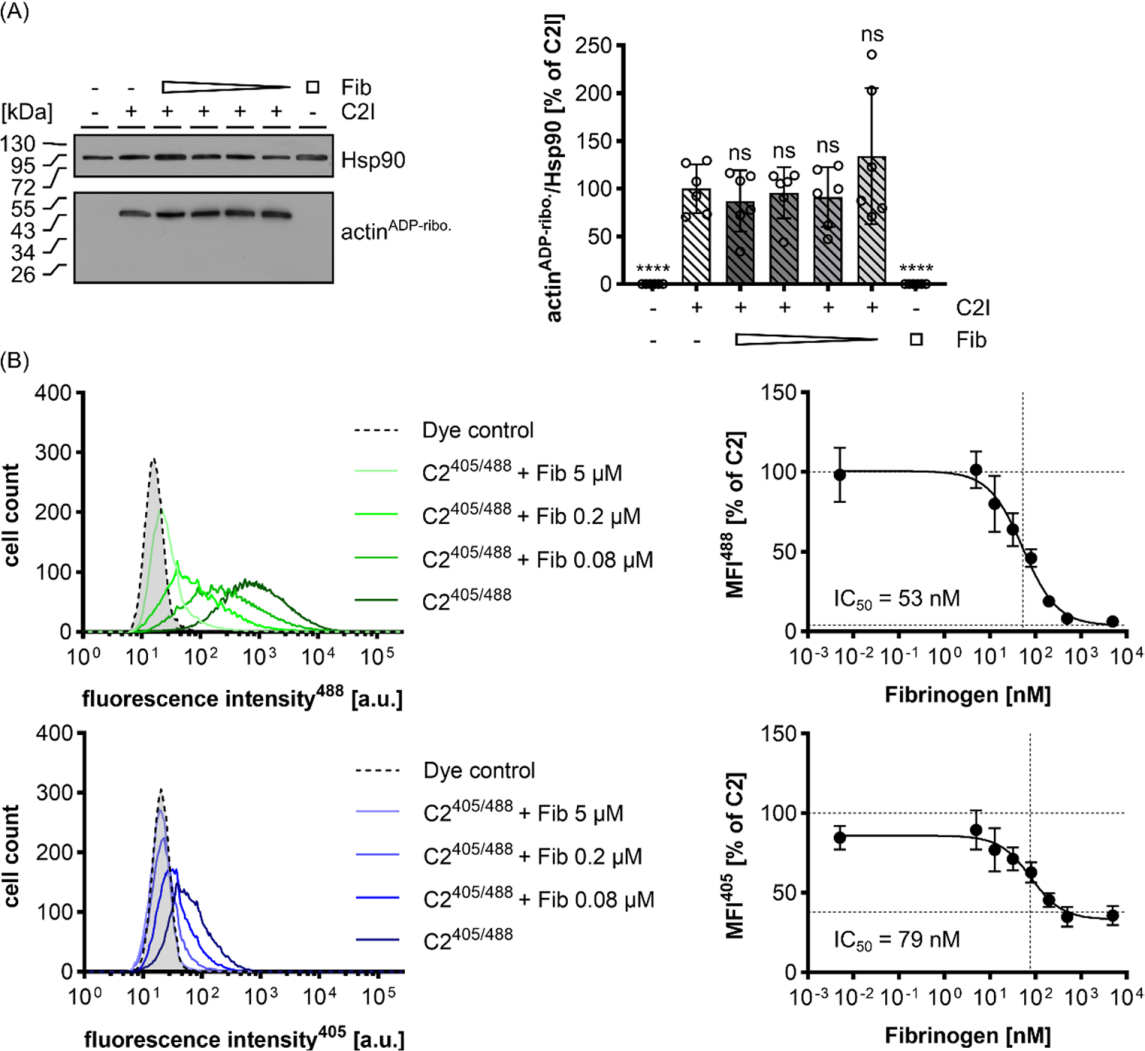
Fib inhibits the binding of C2 to HeLa cells but not the enzyme activity of C2I. (**A**) In vitro enzyme activity assay of C2I. HeLa cell lysate (30 µg) was incubated with Fib (5000 nM, 1000 nM, 200 nM, 40 nM) and C2I (1 nM). Left panel: representative Western blot with ADP-ribosylated actin and Hsp90 as a loading control. Right panel: bar graph showing the quantification of all Western blots. The amount of ADP-ribosylated actin was normalized to the loading control (Hsp90) and to the C2I control. Three individual experiments with duplicates were quantified (*n* = 6). (**B**) Binding assay of C2^405/488^ toxin (C2I^405^ + C2IIa^488^ = C2^405/488^) to HeLa cells. Cells were detached, cooled on ice to prevent toxin uptake and treated with C2I^405^ (16 nM), C2IIa^488^ (13 nM) and Fib as indicated (5000 nM, 500 nM, 200 nM, 80 nM, 32 nM, 12.8 nM, 5 nM and 0.005 nM). Left panel: representative histograms of the fluorescence intensities. Right panel: median fluorescence intensities (MFI) normalized to the C2 control and plotted against the Fib concentration. The horizontal dashed lines represent the mean value of the C2 and dye control for the upper and lower dashed line, respectively, while the vertical dashed line represents the IC_50_ (C2I^405^: 79 nM, C2IIa^488^: 53 nM). Values are given as mean ± SD (*n* = 9) of triplicates from three individual experiments. Statistical analysis was performed compared to the C2 control by using non-parametric one-way ANOVA in combination with Dunnett’s correction for multiple comparison (ns *p* ≥ 0.05, **** *p* < 0.0001)

Although the results obtained from the experiment with C2IIa/H_GTD strongly suggest that Fib interfere with C2IIa function, the effect of Fib on the enzyme activity of C2I was tested by analyzing the in vitro ADP-ribosylation of actin by C2I in the absence and presence of Fib. The results shown in Fig. 5A clearly indicate that Fib has no effect on C2I enzyme activity as the amount of ADP-ribosylated actin in the Western blot was comparable and independent from Fib.

Since inhibition of cytotoxicity by Fib seems to be mediated via C2IIa, we next investigated the binding of C2 toxin to the cell surface. The individual toxin components C2I and C2IIa were labeled with two fluorescent dyes with excitation wavelengths of 405 nm and 488 nm respectively (C2I^405^ + C2IIa^488^ = C2^405/488^). HeLa cells were detached and cooled on ice to stop endocytic processes. The labeled C2^405/488^ was concomitantly applied without Fib or with increasing concentrations of Fib and incubated on ice to allow for toxin binding to the cell surface while preventing uptake. After washing away unbound protein, the remaining cell-bound toxin components were measured by flow cytometry (Fig. 5B). The histograms of the respective fluorescence intensities are shown on the left and median fluorescence intensity (MFI) plotted against Fib concentration on the right side. The upper panel shows fluorescence intensity signals for C2IIa^488^ while the lower panel shows fluorescence intensity signals for C2I^405^. Fib inhibited binding of C2 toxin to cells in a concentration dependent manner and IC_50_ values were calculated at 53 nM and 79 nM for C2IIa^488^ and C2I^405^, respectively. Since C2IIa binding to cells was inhibited by Fib and the IC_50_ values between the two toxin components did not differ much, it is plausible that C2I did not bind to cells because C2IIa was not bound, as it is known that C2I can only bind to cells via C2IIa but not directly [6, 9, 53].

We investigated the binding of C2I and C2IIa to cells and to each other in an alternative approach, where the individual toxin components were added sequentially to the cells with intermediate washing steps. When C2IIa^488^ was applied first to cells followed by Fib and then by C2I^405^, cell-bound C2IIa^488^ was not displaced in the presence of Fib (Supplementary Fig. 3A). However, when Fib was added to cells prior to C2IIa^488^ and then C2I^405^ was added, notably less C2IIa^488^ bound to cells (Supplementary Fig. 3B). Both results suggest that Fib prevents binding of C2IIa to the cell surface. However, these results did not fully clarify whether (i) Fib binds and shields the cellular surface receptor of C2IIa, (ii) dissociates again from the cell and interferes with C2IIa in solution or (iii) captures C2IIa from solution while being bound on the cell surface. Fluorescence signals of C2I were very weak, most likely due to the increase in washing and incubation steps. Since we could not reliably show whether interaction between C2I and C2IIa was hindered in the presence of Fib, we conducted an indirect ELISA. C2IIa was coated to the plate surface and increasing concentrations of Fib were overlayed, followed by a second overlay of a constant concentration of C2I (Supplementary Fig. 3C). Over the whole range of tested Fib concentrations, the same interaction between C2IIa and C2I was observed as in the absence of Fib, indicating that interactions between the A- and B-component is not hindered.

**Fig. 6:**
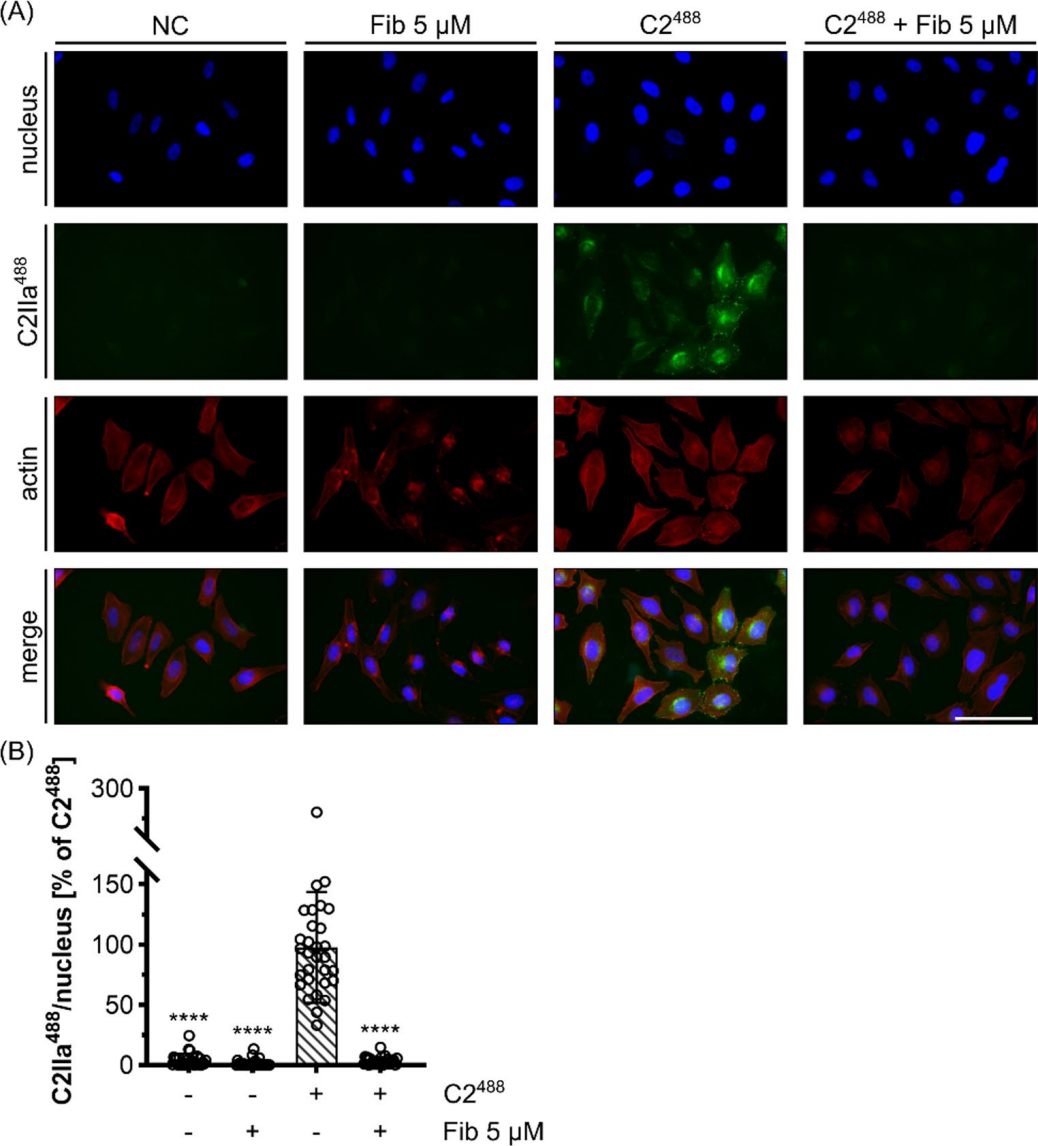
Fib inhibits the binding of C2 toxin to cells and its internalization. (**A**) Representative fluorescence microscopy pictures of HeLa cells treated with Fib (5 µM), C2^488^ (20 nM C2I + 33.2 nM C2IIa^488^) or the combination of Fib and C2^488^. Untreated cells served as a negative control (NC). After 30 min of incubation at 37 °C, cells were fixed and nucleus and F-actin were stained for fluorescence microscopy. Scale bar corresponds to 100 μm. (**B**) Quantification of the C2IIa^488^ signal intensities from (A). C2IIa^488^ was normalized to nuclear signal and the C2^488^ control. Values are given as mean ± SD (*n* = 28–30) of up to ten different areas from three individual experiments. Statistical analysis was performed compared to the C2^488^ control by using non-parametric one-way ANOVA in combination with Dunnett’s correction for multiple comparison (**** *p* < 0.0001)

We used fluorescence microscopy to investigate binding and also cellular uptake of C2 toxin to verify the findings from flow cytometry that Fib inhibits C2IIa binding to cells. Therefore, HeLa cells were treated with C2^488^ (C2I + C2IIa^488^ = C2^488^) in the presence or absence of Fib for 30 min at 37 °C. Under these conditions, cells take up the toxin, which results in cell-associated signals of C2^488^. After the incubation, cells were washed, fixed and stained for F-actin and the nucleus to confirm cell association of C2^488^ signals (Fig. 6A). An almost complete reduction of C2^488^ signal was observed when Fib was present, supporting the flow cytometry results. C2^488^ signal was quantified and normalized to nuclear signal to correct for cell density between pictures (Fig. 6B), which confirmed that Fib inhibits binding of C2IIa and thereby also uptake of C2 toxin. In order to exclude that Fib is activated to fibrin within the first hour, i.e. when binding and internalization of C2 toxin occurs, ROTEM experiments were performed in parallel (Supplementary Fig. 4). The results showed that Fib remains a precursor protein, implying that it is indeed Fib and not fibrin that shows C2 toxin-inhibiting capacity.

To confirm this key finding and to demonstrate the specificity not only of the inhibition but also of the assays used, we compared the influence of Fib on C2IIa vs. Ib (B-component of iota) binding to Vero cells. Vero cells were chosen because both toxin receptors are expressed in this cell line. Toxin binding of fluorescently labelled C2IIa^488^ and Ib^488^ to the cell surface was measured by flow cytometry (Supplementary Fig. 5). In the presence of 5 µM Fib, binding of C2IIa^488^ to the cells was significantly reduced (Supplementary Fig. 5A) while the binding of Ib^488^ to the cells was comparable in the absence and presence of Fib (Supplementary Fig. 5B).

### Fib directly binds to C2IIa *in vitro*

**Fig. 7:**
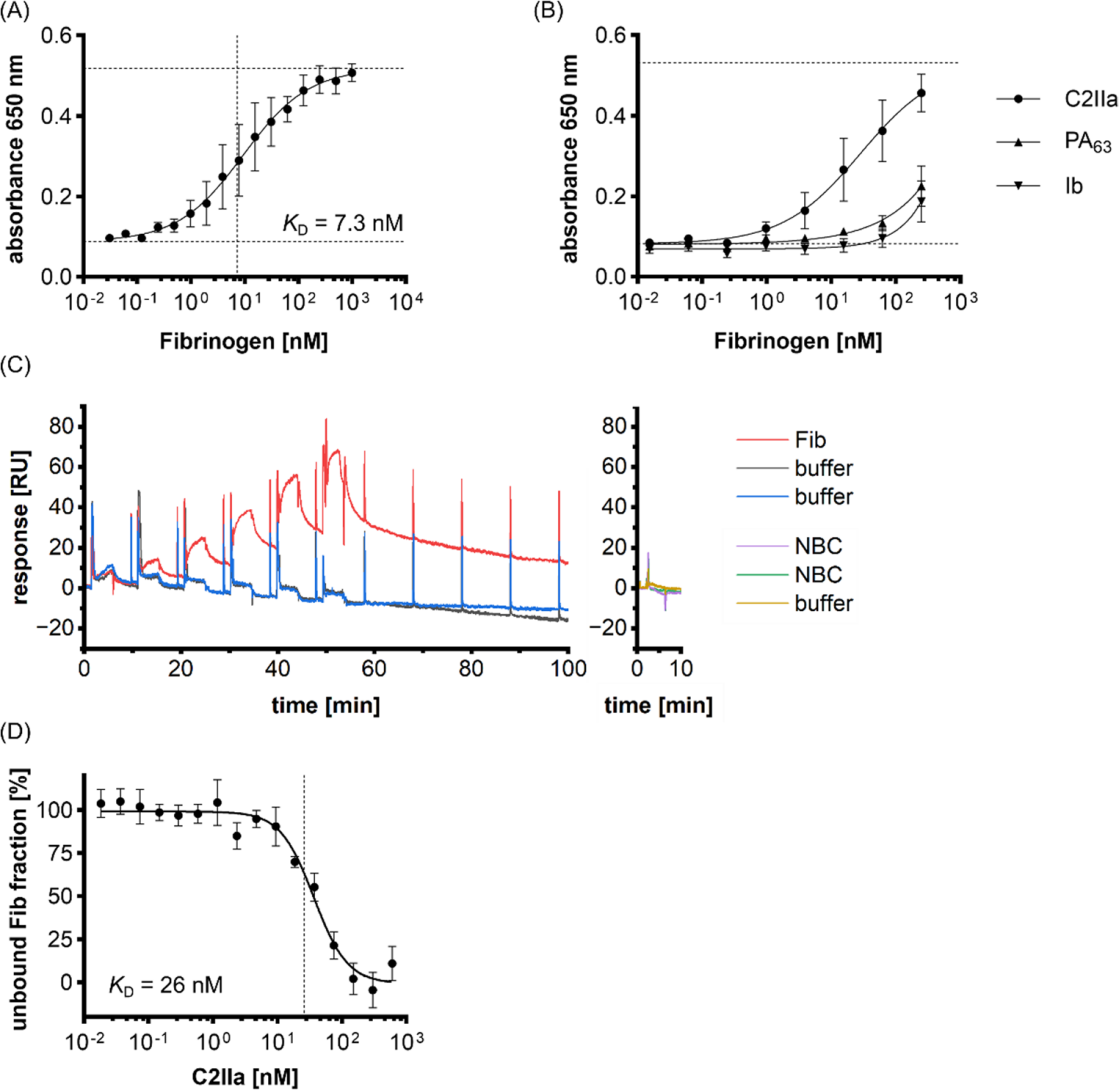
Fib directly interacts with C2IIa but not PA_63_ or Ib. (**A**) Titration ELISA with C2IIa coated to the plate and a sequential 1:2 dilution series of Fib (1000 nM to 0.03 nM). Values are given as mean ± SD (*n* = 8) of duplicates from four individual experiments. Horizontally dashed lines indicate upper and lower limit of the applied fit while the vertical dashed line is at *K*_D_ = 7.3 nM calculated after Eble JA [54]. (**B**) Titration ELISA with C2IIa, PA_63_ or Ib coated to the plate and an overlay of Fib. Respective toxin components were coated and Fib was added in a sequential 1:4 dilution series (250 nM to 0.015 nM). Values are given as mean ± SD (*n* = 8) of duplicates from four individual experiments. Horizontally dashed lines indicate upper and lower limit the applied fit for C2IIa and Fib interaction. (**C**) Single cycle kinetic SPR measurement of the binding of Fib to 1800 RU of immobilized C2IIa (left graph). Increasing concentrations of Fib (39 nM; 156 nM; 625 nM; 2.5 µM; 10 µM and 40 µM) were applied for 4 min each followed by a dissociation phase for 5 min and a final dissociation phase for 60 min. Buffer measurements are included as control in the same graph. NBC (non-binding control) injections (10 µM of FH15-19) on the same chip confirm specificity of binding (right graph) (**D**) Binding affinity measurement of labelled Fib with C2IIa in solution. C2IIa was used in a sequential 1:2 dilution series (600 nM to 0.018 nM C2IIa) and Fib with a constant concentration (20 nM). Values are given as mean ± SD (*n* = 3) from three individual experiments. Vertically dashed line is at *K*_D_ = 26 nM as calculated with the MO.Control 2.6.3 software

The cell-based experiments revealed a robust inhibition of C2IIa binding to cells by Fib, but they gave no hint whether this inhibition was due to a block of the cell surface receptor or caused by a direct interaction of Fib with C2IIa. To test the latter possibility, C2IIa was coated to ELISA plates and binding of Fib was measured via titration ELISA (Fig. 7A, for controls compare Supplementary Fig. 6A). A concentration-dependent binding of Fib to the immobilized C2IIa was measured with a *K*_D_ = 7.3 nM (calculated after Eble JA [54]). Neither PA_63_ nor Ib showed a comparable interaction with Fib in this approach (Fig. 7B, for controls compare Supplementary Fig. 6B). In order to confirm the results obtained from ELISA experiments by an alternative approach, we performed SPR experiments (Fig. 7C). C2IIa was immobilized by covalent lysin linkage to the SPR sensor chip and Fib was injected. Due to the heptameric nature of C2IIa, chip regeneration in between cycles was avoided to limit disturbance or even denaturation of the formed heptameric complex. Of note, already during continuous flow with running buffer, a very slow but constant reduction of the response signal was observed, indicating a loss of C2IIa on the chip surface, most likely due to depolymerization of the C2IIa heptamer (more detailed description within the materials and methods section). As shown in Fig. 7C, a concentration-dependent interaction between C2IIa and Fib was detected, confirming the results of the ELISA experiments. The NBC did not show interaction with C2IIa and additionally did not show any biological effect regarding C2 inhibition (Supplementary Fig. 7). These results were confirmed by a repetition of the Fib single cycle kinetic experiment on a second chip supplemented with a multi cycle like experiment and repeated injections of the same concentration of Fib (Supplementary Fig. 8). Moreover, binding affinity experiments without immobilization of one binding partner supported previous results and showed that labelled Fib directly interacts with C2IIa in solution with a *K*_D_ = 26 nM (Fig. 7D). As three different methods showed direct interaction between Fib and C2IIa and two methods yielded *K*_D_ values in an overall comparable range (*K*_D_ = 7.3 nM and 26 nM), these results strongly suggest that the inhibition of C2 toxin by Fib is mediated by direct protein interaction.

### Human plasma-derived Fib binds to C2IIa *ex vivo*

**Fig. 8:**
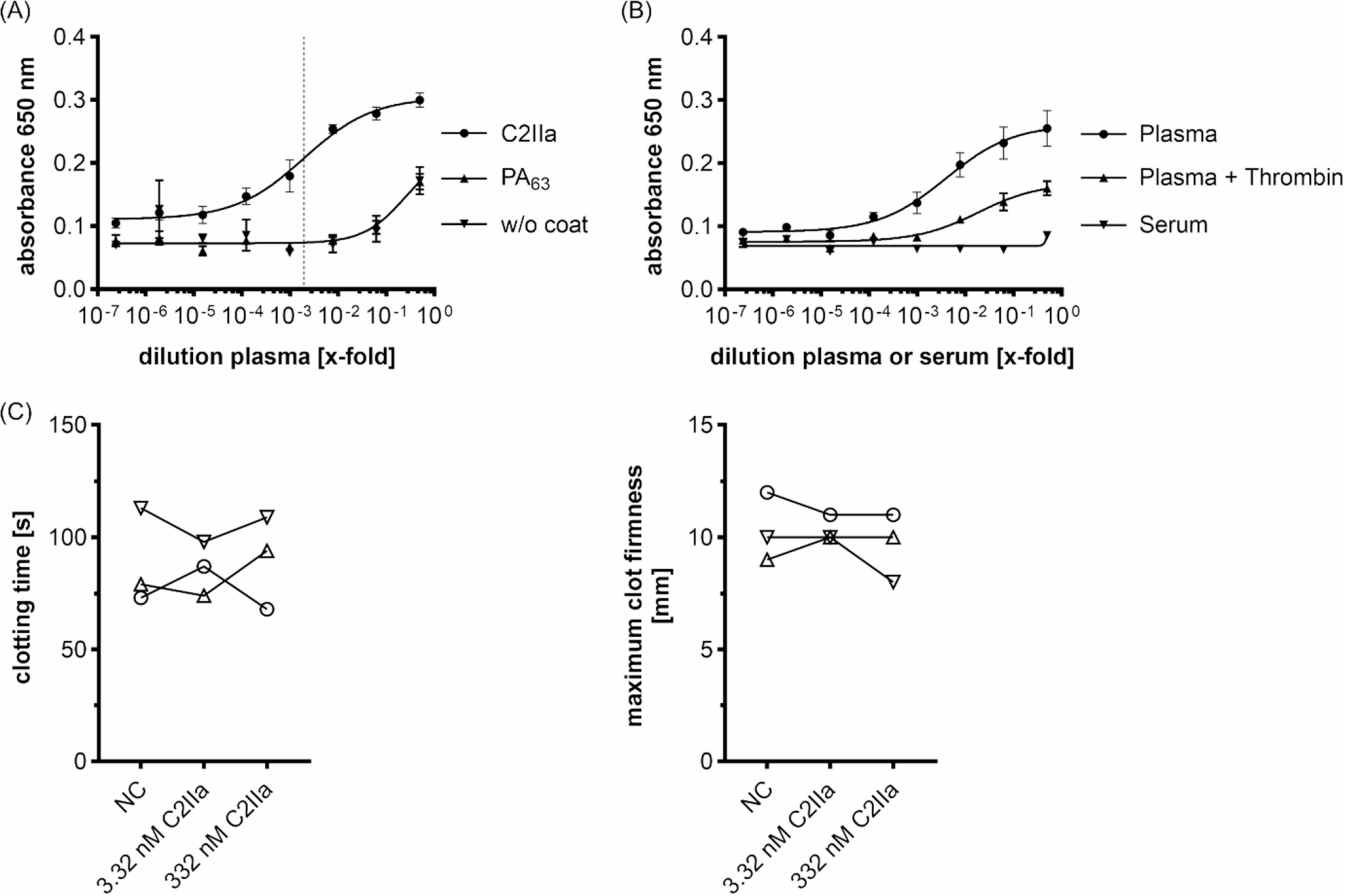
Plasma-derived Fib interacts with C2IIa but not with PA_63_. (**A**) ELISA of coated C2IIa and human plasma as overlay. C2IIa, PA_63_ or PBS was coated to the plate and plasma was added in a sequential 1:8 dilution starting from a 1:2 dilution. PA_63_ and C2IIa are given as mean ± SD (*n* = 6) of duplicates, plasma only values are given as mean ± SD (*n* = 3) of single measurements from three individual experiments. The vertical dashed line is at EC_50_ = 1.957 × 10^-3^ c(plasma) for C2IIa and plasma interaction. (**B**) ELISA of coated C2IIa and human plasma, thrombin activated plasma or serum as overlay. C2IIa was coated to the plate and either plasma, thrombin activated plasma, or serum was added in a sequential 1:8 dilution starting from a 1:2 predilution. Values are given as mean ± SD (*n* = 6) of duplicates from three individual experiments. (**C**) C2IIa has no influence on the Fib-associated hemostatic system parameters of whole blood. FIBTEM measurements of whole blood (NC) and whole blood supplemented with the indicated concentrations of C2IIa. The coagulation parameters clotting time (left graph) and maximum clot firmness (right graph) are depicted. Symbols correspond to samples from individual donors (*n* = 3)

Finally, the results were confirmed with Fib directly obtained from fresh human blood plasma of healthy donors. In a first experiment, C2IIa or PA_63_ were coated to ELISA plates and human plasma samples were added in a sequential dilution series (Fig. 8A, for controls compare Supplementary Fig. 9A). Consistent with previous ELISA results, a concentration-dependent binding to C2IIa, but not to PA_63_ was measured, suggesting specific binding of Fib from the plasma to C2IIa. For further evidence, the same experiment was performed with immobilized C2IIa and overlaid with either human plasma, human plasma activated with thrombin (plasma + thrombin), or human serum (Fig. 8B, for controls compare Supplementary Fig. 9B), which were confirmed to contain less or no Fib respectively (Supplementary Fig. 9C, D). Again, Fib within the plasma bound to C2IIa whereas serum (Fib depleted) showed no binding as expected. Plasma after Fib depletion by thrombin cleavage showed intermediate results indicating an incomplete Fib activation/removal. The residual Fib levels were determined in parallel via ELISA and Western blot (Supplementary Fig. 9C, D), explaining the slight interaction of activated plasma with thrombin and C2IIa. In conclusion, Fib in human blood binds to and inhibits C2IIa ex vivo, confirming physiological relevance of this observation.

Moreover, we investigated whether the presence of C2IIa in human blood may influence blood coagulation because it binds to Fib, thereby sequestering this central coagulation factor. To this end, we analyzed Fib associated hemostatic system parameters of whole blood in the absence and presence of C2IIa (Fig. 8C, D). Neither low nor high C2IIa concentrations did affect the clotting time or firmness of the clot evaluated by ROTEM analysis. These results indicate that the toxin-neutralizing effect of Fib does not negatively affect the physiological functions in terms of coagulation.

### N-glycosylation of Fib is essential for binding to C2IIa

**Fig. 9:**
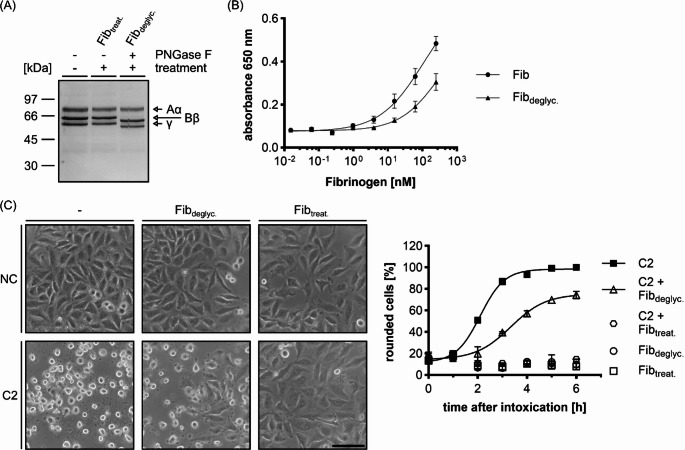
The N-glycosylation of Fib is essential for the inhibitory effect against the C2 toxin. (**A**) SDS-PAGE and Coomassie staining of Fib samples before and after deglycosylation. Fib was left untreated (first lane) or treated with or without PNGase F (second and third lane) for 75 min at 37 °C. (**B**) ELISA of coated C2IIa and Fib or Fib_deglyc_. as overlay. C2IIa was coated to the plate and Fib samples were added in a sequential 1:4 dilution (250 nM **− 0**.015 nM). Values are given as mean ± SD (*n* = 6) of duplicates from three individual experiments. (**C**) Left panel: representative pictures of HeLa cells after 6 h with 0.35 µM Fib_deglyc_. or Fib_treat_. in the presence or absence of C2 toxin (2 nM C2I + 3.32 nM C2IIa). Untreated cells served as a negative control (NC). Scale bar corresponds to 100 μm. Right panel: quantification of the C2 toxin-induced cell rounding from the same experiment. Pictures were taken at the indicated time points and the percentage of rounded cells from the total cell number was quantified. Values are given as mean ± SD (*n* = 2) of duplicates from one representative experiment

Based on the observations that Fib directly interacts with C2IIa and thereby prevents its binding to the cell surface, we investigated whether N-glycosylation patterns on the surface of Fib mediate the interaction with C2IIa. Fib is post translationally N-glycosylated at its Bβ and γ chain [55], which may mimic cellular asparagine-linked carbohydrates that are essential for C2IIa binding to cells [11]. Therefore, N-linked glycans were removed from Fib using PNGase F under mild reactions conditions (37 °C, without denaturing agents) and the resulting protein (Fib_deglyc_.) was analyzed by SDS-PAGE and Coomassie staining (Fig. 9A). The Bβ and γ chains showed deglycosylation after treatment with PNGase F, which is apparent by a molecular weight shift, while the naturally non-glycosylated Aα chain showed no shift in molecular weight as expected. To test whether the N-linked glycans of fibrinogen are important for the interaction, C2IIa was coated to ELISA plates and both variants of Fib were used as an overlay. Fib_deglyc_. showed lower affinity towards C2IIa in the ELISA setting as observed by a right shift of the curve (Fig. 9B, for controls compare Supplementary Fig. 10). The lower affinity of Fib_deglyc_. was verified using a cytotoxicity assay with HeLa cells (Fig. 9C). Here, Fib_deglyc_. only delayed the C2 intoxication, as compared to a complete inhibition of Fib that was subjected to the same treatment but without the addition of PNGase F enzyme (Fib_treat_.). Taken together, these findings indicate that the N-linked glycans on Fib are crucial for the binding to C2IIa and are therefore necessary for the inhibitory capacity of Fib against C2 toxin.

## Discussion

Recently we have introduced the concept of “trauma-toxicology” addressing the interplay between the thrombo-inflammatory response and associated exposure to endogenous and exogenous toxins following physical injury [2]. The coagulation cascade thus not only exerts hemostatic functions but also immunomodulatory effects [38, 39], which prompted us to investigate the interplay between bacterial protein toxins and this system. As a central downstream component of the coagulation system, Fib is critical to prevent excessive blood loss after injury [56, 57]. Structurally, Fib is a glycoprotein with a molecular weight of approximately 340 kDa and consists of three pairs of polypeptide chains (Aα, Bβ, and γ chains) that are linked by disulfide bonds. It is produced in the liver and circulates in its inactive form in the blood plasma [56, 58, 59]. Fib is post-translationally modified at specific asparagine residues of the Bβ and γ chains, which is also essential for proper fibrin polymerization and clot architecture [55, 60]. During coagulation, Fib acts as a bridging molecule essential for the aggregation of activated platelets during the initial phase of blood clot formation. Upon activation of the blood coagulation cascade, thrombin cleaves specific peptide bonds in the Fib molecule, activating Fib, which then assembles into insoluble fibrin [57, 61]. The resulting fibrin strands aggregate and the fibers crosslink to form a stable clot at the site of injury [57, 62]. In addition to its primary role in hemostasis, Fib also plays an essential role in inflammation, tissue repair, and wound healing [56, 57].

In the present study, we identified Fib as a potent inhibitor of the binary C2 toxin from *C. botulinum*. This is the first description of a blood coagulation factor neutralizing a bacterial AB-type protein toxin in a highly specific manner. We further investigated the molecular mechanism underlying this toxin-inhibiting effect and found that Fib inhibits C2 toxin binding to cells by directly binding to the toxins B-subunit C2IIa. Extracellular interaction with the toxin is also the most plausible type of inhibition for protein inhibitors due to the lack of cell penetration. This mode of toxin inhibition was also observed by us and others for other body-own proteins and peptides, including such from human blood [24, 26–31, 35, 36, 63, 64].

Other bacterial exotoxins were analyzed to determine a transferability of Fib´s inhibitory effect to other toxins. Our results demonstrate that Fib specifically and strongly inhibits C2 toxin but not closely related binary toxins of the same family including *Clostridioides difficile* CDT, *C. perfringens* iota toxin or *Bacillus anthracis* anthrax toxin. The inhibition is not mediated via a direct interaction between Fib and C2I, since Fib neither inhibit C2I’s enzyme activity nor disrupted the interaction between C2I and C2IIa. This is further supported by the fact that the other actin ADP-ribosylating toxins CDT and iota were also not inhibited by Fib, with both toxins A-subunits being structurally and functionally highly related to C2I [50, 65]. However, the transport of a His-tagged foreign enzyme into cells by C2IIa [44] was inhibited by Fib, confirming the hypothesis that the specificity of the inhibitory effect is mediated by the interaction of Fib with C2IIa.

In line with the observations that C2I functions were unaffected, we found a specific interaction between Fib and C2IIa in several approaches. Although the exact binding site of Fib on C2IIa remains undetermined, our results demonstrate that the C2I/C2IIa interaction is unaffected, indicating that the binding site for C2I within C2IIa is not sterically hindered. Hence, D1’ in C2IIa is most likely not bound by Fib [43]. Domain 1 (D1) is located at the N-terminus of C2IIa and contains the site for proteolytic activation, after which a part of D1 (D1’) remains on the now activated C2IIa for binding of C2I [50, 66]. Since the B-subunits of C2 toxin, iota toxin, CDT, and anthrax toxin share a heptameric structure and function as pore-forming transport proteins for their A-subunits, Domains 2 and 3 are related between the proteins and exhibit similar functions [50, 66, 67]. The main difference in both sequence and function are the C-terminally located receptor-binding domains (D4) [43, 68]. While Ib and CDTb are more similar to each other and bind the same cellular receptor protein, namely LSR [51], both PA_63_ and C2IIa share low sequence and structure homology in D4 compared to Ib and CDTb and bind to different receptors. PA_63_ binds to TEM8/ANTXR1 or CMG2/ANTXR2 [69, 70]. Hence, D4 appears to be the most likely candidate for interaction between C2IIa and Fib. This is also in line with our results that C2IIa was no longer able to bind to cells in the presence of Fib, possibly implicating a steric hinderance of D4 or an induced conformational change that renders C2IIa non-binding. Our experiments revealed, that the N-linked glycans of Fib are essential for the interaction with C2IIa and the inhibition of C2 toxin mediated cytotoxicity. These findings further hint towards D4 being responsible for the interaction between C2IIa and Fib, as C2IIa naturally binds complex and hybrid N-linked glycan structures on the cell surface [11]. The N-linked glycosylation of Fib on the Bβ and γ chains, might thus mimic the cellular receptor of C2IIa and act as a soluble kind of decoy receptor in the blood. To directly prove this hypothesis, we tried to recombinantly express D4 of C2II as an isolated protein. However, the purified D4 did not bind to cells, as determined by missing competition against C2IIa, indicating that this purified D4 protein was biologically inactive, most likely caused by misfolding or missing oligomer formation. This is in line with previous results that the recombinant production of the individual domains of C2II yields biologically inactive proteins [68]. We further cloned chimeric fusion proteins in which D4 was exchanged between C2II and PA_83_ (protective antigen before proteolytic activation to PA_63_). Also, these two purified proteins were biologically inactive (despite the presence of domains 1–3), as evident by their missing ability to intoxicate cells, when combined with their respective A-subunits (unpublished data). C2II from the *C. botulinum* strain 2300 could be used to further investigate the function of D4, as this version contains an C-terminal extension of 129 amino acids, that presumably resulted from a duplication of this domain. As this version shows higher affinity towards the cellular receptor, also the interaction with Fib might be enhanced [71].

Taken together, our data reveal a novel biological function of human Fib, namely neutralization of a bacterial AB-type protein toxin by direct and specific binding to its B-subunit. Fib prevented toxin binding to and therefore uptake into cells, protecting cells from intoxication. Notably, the ability of Fib to aggregate in an ex vivo whole human blood model was not impaired in the presence of C2IIa, indicating no adverse effect on hemostasis by the toxin-neutralizing effect. This could be expected due to the abundance of Fib in human plasma. Fib was also not aggregated in vitro by C2IIa and aggregation, i.e. formation of fibrin, was only observed with canonical proteolytic activation by thrombin. Moreover, the results implicate that Fib and not its activated form, fibrin, exhibits inhibitory capacity against C2 toxin. In the clinical context, however, it would be essential to balance the extent to which Fib supports innate immune defense through toxin neutralization against the potential for Fib-mediated binding of toxins and their subsequent potential incorporation into clots or healing tissues, which may promote chronic inflammation and fibrotic tissue remodeling. This first report of a blood coagulation factor neutralizing a highly potent bacterial exotoxin implicates that human Fib has additional non-canonical functions in the defense against bacterial protein toxins and expands current knowledge of Fib in the context of innate immunity.

## Materials and methods

### Toxins and reagents

The recombinant components C2I and C2II from *Clostridium botulinum* C2 toxin (originally cloned from C. botulinum strain KZZ1577 (92 − 13) were expressed, purified and activated as described earlier (C2I: [8]; C2IIa: [68, 72]). PA_63_ from *Bacillus anthracis* was purified and activated as mentioned elsewhere [73, 74]. The His_6_-tagged glucosyltransferase domain of *C. difficile* (H_GTD) was purified as described earlier [44]. LF_N_-DTA was kindly provided by R. J. Collier (Boston, MA, USA), iota toxin components by M. R. Popoff (Paris, France), CDT components and TcdB by K. Aktories (Freiburg, Germany).

The C2 toxin components C2I and C2IIa were labeled with different dyes (C2I: DyLight™ 405 NHS Ester; C2IIa: DyLight™ 488 NHS Ester, both from Thermo Fisher Scientific, Waltham, MA, USA). The labeling was performed according to the manufacturer’s instructions. To remove unbound dye Zeba™ Spin Desalting Columns (7k MWCO, Thermo Fisher Scientific, Waltham, MA, USA) were used. The corresponding dye controls contained PBS (137 mM NaCl, 2.7 mM KCl, 8 mM Na_2_HPO_4_, 1.8 mM KH_2_PO_4_; pH 7.4) with the equal amount of dye used to label the toxin. The dye controls were desalted the same way.

For dye control of Ib^488^ a small volume of labeled toxin was centrifuged in a Vivaspin^®^ centrifugal concentrator 5 kDa MWCO (Sartorius, Goettingen, Germany) and the flowthrough containing unbound dye was used.

Fibrinogen (Sigma-Aldrich, St. Louis, MO, USA) and Camptothecin (Thermo Fisher Scientific, Waltham, MA, USA) were purchased. For binding affinity experiments fibrinogen was labeled with the Protein Labeling Kit RED-NHS 2nd Generation (NanoTemper, Munich, Germany) according to the manufacturer’s instructions.

Factor H fragment 15–19 (FH15-19) was purified as described earlier [75, 76].

### Cell culture and cytotoxicity assay

HeLa cells and Vero cells were cultured in MEM medium (Gibco-Life Technologies, Carlsbad, CA, USA) supplemented with 10% fetal calf serum (Gibco-Life Technologies, Carlsbad, CA, USA), 1% sodium pyruvate (Gibco-Life Technologies, Carlsbad, CA, USA), 1% L-Glutamine (PAN-BIOTECH, Aidenbach, Germany), 1% MEM-NEAA (Gibco-Life Technologies, Carlsbad, CA, USA), and 100 U/mL (1%) penicillin-streptomycin (Gibco-Life Technologies, Carlsbad, CA, USA). Both cell types were sub-cultivated three times per week 1:3 or 1:10 after trypsinization (PAN-BIOTECH, Aidenbach, Germany). CaCo-2 cells were cultured in DMEM medium (Gibco-Life Technologies, Carlsbad, CA, USA) supplemented with 10% fetal calf serum, 1% sodium pyruvate and 100 U/mL (1%) penicillin-streptomycin and sub-cultivated 1:2 or 1:5 three times per week after trypsinization. All cells were cultured at 37 °C and 5% CO_2_ with saturated humidity and imaged using either an Axiovert 40CFL microscope (Zeiss, Jena, Germany) with a ProgRes C10 CCD camera (Jenoptik, Jena, Germany) or a Leica DMi1 microscope with a Leica MC170 HD camera (both Leica, Wetzlar, Germany).

For cytotoxicity assays, the respective growth medium was replaced by medium containing the toxins in the presence or absence of the inhibitor Fib as indicated in the individual experiment. The medium itself was prepared with serum-free medium for HeLa cells and Vero cells and with serum-containing medium for CaCo-2 cells. Cells were imaged at the indicated time points and subsequently quantified based on their morphology using neuralab.de software (Neuralab, Ulm, Germany). Quantification via Neuralab software was verified for each picture. For graphical representation of cytotoxicity kinetics, a sigmoidal 4PL fit was applied with GraphPad Prism. Statistical analysis was performed as mentioned later and multiple comparison test was performed against the toxin condition to test for significant inhibition.

HeLa (human cervix carcinoma cells; DSMZ, Braunschweig, Germany), Vero (African green monkey kidney cells; DSMZ, Braunschweig, Germany) and CaCo-2 (human colorectal adenocarcinoma cells; HTB-37, ATCC, Manassas, VA, USA) cells were all commercially obtained.

### SDS-PAGE and Western blotting

Electrophoretic separation of proteins was performed via SDS-PAGE using 6% stacking gels and 12.5% running gels. All samples were prepared with 5x Laemmli (0.3 M Tris-HCl, 10% SDS, 37.5% glycerol, 0.4 mM bromophenol blue, 100 mM DTT) and denatured at 95 °C for 10 min. After separation in SDS running buffer (0.1% SDS, 27.2 mM Tris-HCl, 192 mM glycine) proteins were transferred onto a nitrocellulose membrane (Cytiva, Marlborough, MA, USA) via semi-dry blot using Towbin blot buffer (20% MeOH, 27.2 mM Tris-HCl, 192 mM glycine). The membrane was then stained with Ponceau S (AppliChem GmbH, Darmstadt, Germany) to determine total protein loading and to verify blotting efficacy before blocking with 5% skim milk powder (Carl Roth, Karlsruhe, Germany) solution in PBS-T (PBS + 0.1% Tween-20) for 1 h at room temperature. The membrane was incubated either with streptavidin-peroxidase conjugate (1:5000 in PBS-T; Sigma-Aldrich, St. Louis, MO, USA) for detection of biotin-labeled proteins or with consecutive incubations of primary and secondary antibody overnight at 4 °C or for 1 h at room temperature. Hsp90 α/β antibody (F-8) (1:1000 in PBS-T, Santa Cruz Biotechnology, Dallas, TX, USA), goat anti-Mouse IgG (H + L) HRP conjugate (1:2500 in PBS-T; Thermo Fisher Scientific, Waltham, MA, USA) and ALB/Albumin Antibody (F-10) conjugated to Alexa Fluor^®^ 488 (1:500 in PBS-T, Santa Cruz Biotechnology, Dallas, TX, USA) were used. Peroxidase was detected by Pierce ECL western blotting substrate (Thermo Fisher Scientific, Waltham, MA, USA). The chemiluminescence signal was detected using a dark chamber with medical X-ray films (AGFA Health Care, Mortsel, Belgium) or the iBright™ CL1500 Imaging System (both Thermo Fisher Scientific, Waltham, MA, USA). The iBright™ was also used to detect fluoroblots.

### Probing the intracellular actin ADP ribosylation status of intact HeLa cells

1.2 × 10^5^ HeLa cells per well were seeded into a 48-well microtiter plate. After two days, cells were treated with C2 in the presence or absence of Fib as indicated in the individual experiment. 24 h later, cells were scraped out, washed with PBS and resuspended in 20 µl ADP-ribosylation buffer (20 mM Tris–HCl (pH 7.6), 1 mM EDTA, 1 mM DTT, and 5 mM MgCl_2_) containing cOmplete™ protease inhibitor (Roche, Basel, Switzerland). To analyze the intracellular ADP-ribosylation status of actin, cells were transferred to the post-ADP-ribosylation reaction in which the remaining amount of non-ADP-ribosylated actin was ADP-ribosylated and thereby biotinylated. Therefore, C2I (6 pmol) and Biotin-NAD^+^ (250 pmol, R&D Systems, Minneapolis, MN, USA) were added, and the cells were lysed for at least 1 h at -20 °C. The enzymatic reaction followed at 37 °C for 30 min and was stopped by adding 5x Laemmli buffer. Further analysis occurred via SDS-PAGE and Western blot. Quantification was carried out with ImageJ (v1.52f, National Institute of Health, Bethesda, MD, USA) and the results were normalized to the total protein amount and negative control (w/o C2, w/o Fib). Statistical analysis was performed as mentioned later and multiple comparison test was performed against the toxin condition (w/ C2, w/o Fib) to test for significant inhibition.

### Cell viability assay

CellTiter 96^®^ AQueous One solution containing 3-(4,5-dimethylthiazol-2-yl)-5-(3-carboxymethoxyphenyl)-2-(4- sulfophenyl)-2H-tetrazolium (MTS) (Promega, Madison, WI, USA) was used to determine the cell viability of HeLa cells. 6 × 10^3^ cells per well were seeded into 96-well microtiter plates and treated under serum containing conditions as indicated in the individual experiments. MTS was added to each well equivalent to 10% of the total well volume at the indicated time points and incubated for 1 h at 37 °C and 5% CO_2_ content. Absorbance measurements were performed afterwards using the TriStar^2^ LB 942 microplate reader (Berthold Technologies GmbH & Co.KG, Bad Wildbad, Germany) at a wavelength of 492 nm. For each timepoint absorbance values were normalized to negative control, giving the relative cell viability. Statistics were always performed against C2 condition within the individual timepoints to show significant inhibition.

### Apoptosis assay via flow cytometry

2 × 10^4^ HeLa cells per well were seeded into 24-well microtiter plates and treated as indicated two days after cell seeding. 24 h after treatment the cells were detached by trypsin/EDTA (PAN-BIOTECH, Aidenbach, Germany) after washing with PBS. All volumes (medium from treatment, PBS wash and trypsin/EDTA detach) were pooled to collect also detached cells, centrifuged, washed with PBS and resuspended in 100 µl Annex-V-Buffer (10 mM HEPES, 140 mM NaCl, 2.5 mM CaCl_2_, pH 7.4) containing 1:20 Annexin-V-Alexa Fluor 488 conjugate (Annexin-V) and 1:100 SYTOX™ Blue (Sytox) (both Thermo Fisher Scientific, Waltham, MA, USA). After 15 min, another 100 µl Annex-V-Buffer was added and the fluorescence intensity of the cells measured by flow cytometry at 488 nm and 405 nm with the BD FACSCelesta™ Cell Analyzer (Becton, Dickinson and Company, Franklin Lakes, NJ, USA). BB515 and BV421 filters were used for 488 nm and 405 nm detection, respectively. Analysis of the raw data was carried out using Flowing Software 2.5.1 (Turku Bioscience, Turku, Finland) and GraphPad Prism was used for graphical representation.

### Fluorescence microscopy

3 × 10^3^ HeLa cells per well were seeded into 18-well µ-slides (ibidi GmbH, Gräfelfing, Germany) and cultured for two days. Afterwards, medium containing C2 or fibrinogen was prepared as indicated in the individual experiment. Before the medium was added to the cells for 30 min at 37 °C and 5% CO_2_ content, it was centrifuged to precipitate aggregated protein. Cells were washed with PBS and fixed with 4% paraformaldehyde (PFA) (diluted in PBS; Merck, Darmstadt, Germany) for 20 min at room temperature. The actin cytoskeleton was stained with SiR-actin (1:2000 in PBS; Spirochrome AG, Stein am Rhein, Switzerland) for 45 min at room temperature. Cells were washed with PBS-T (PBS + 0.1% Tween-20) and the nucleus was stained with Hoechst 33,342 (1:5000 in PBS; Thermo Fisher Scientific, Waltham, MA, USA) for 5 min at room temperature. Cells were washed with PBS-T and PBS before imaging with the Keyence BZ-X800 series fluorescence microscope (Keyence, Osaka, Japan) with different filter cubes depending on the stained cellular structure (actin: BZ-X Filter Cy5 (OP-87766), C2IIa^488^: BZ-X filter GFP (OP-87763), nucleus: BZ-X filter DAPI (OP-87762)). Fluorescence intensity signals (signals) form the GFP channel (C2IIa^488^) and the DAPI channel (Hoechst33342) were quantified using ImageJ. Therefore, images were converted to an 8-bit image and a histogram for pixel grey values was generated. After background subtraction (based on gray values of areas without a specific signal), the pixel counts from the histogram was multiplied with the corresponding adjusted grey value (background subtracted) and values for all pixels of the image were summed up to yield the fluorescence intensity signal for a respective channel in an image. GFP signal was normalized to DAPI signal (normalization for cell density in the picture) and then normalized to C2^488^ control. In total 28–30 pictures of three independent experiments were quantified excluding obvious outliers (i.e. pictures with extracellularly aggregated and not cell associated fluorescent protein).

6 × 10^4^ CaCo-2 cells per well were seeded into 8-well µ-slide (ibidi GmbH, Gräfelfing, Germany). After two days, the growth medium was replaced by medium containing C2 with or without fibrinogen as indicated in the individual experiment and incubated for 5. Cells were washed with PBS and fixed with 4% PFA as mentioned above. The actin cytoskeleton was stained with SiR-actin and the cell membrane with Lectin from *Triticum vulgaris*-FITC (1:100 in PBS; Sigma- Aldrich, St. Louis, MO, USA) for 45 min at room temperature. Nucleus staining, washing steps and fluorescence microcopy were performed as described for HeLa cells using the Keyence BZ-X800 series fluorescence microscope with the respective filter cubes (actin: BZ-X Filter Cy5, cell membrane: BZ-X filter GFP, nucleus: BZ-X filter DAPI).

### *In vitro* enzyme activity of C2I

HeLa cell lysate (30 µg) was incubated with C2I (1 nM), Biotin-NAD+ (10 µM) and fibrinogen as indicated in the individual experiment in a total volume of 25 µl ADP-ribosylation buffer. The whole reaction was incubated for 30 min at 37 °C and stopped by adding 5x Laemmli. SDS-PAGE and Western blot were performed as mentioned before. Quantification was carried out with ImageJ and the results were normalized to the loading control and C2I control (w/ C2I, w/o Fib). Statistical analysis was performed as mentioned later and multiple comparison test was performed against the toxin condition (w/ C2I, w/o Fib) to test for significant inhibition.

### Transepithelial electrical resistance (TEER) assay

1.5 × 10^5^ CaCo-2 cells were seeded into BRAND Inserts for 24- well plates (BRAND GmbH & Co.KG, Wertheim, Germany) and the surrounding well was filled with growth medium. Cells were cultured at 37 °C and 5% CO_2_ until TEER values of at least 1000 ohms were reached. Then, medium with 7x higher concentrations of C2 or fibrinogen was prepared and added to the medium to reach the final concentrations as mentioned in the individual experiment. TEER measurements were performed using the Millicell^®^ ERS-2 Voltohmmeter (Merck, Darmstadt, Germany) and the resistance values were normalized to the corresponding 0 h time point. Individual measurements were connected by a line for graphical representation.

### Analysis of toxin binding to the cell surface

HeLa cells or Vero cells were cultured for two to three days in 10 cm dishes. Cells were detached mechanically after a 20 min incubation with 25 mM EDTA at 37 °C and 5% CO_2_. Cells were collected, washed with PBS and resuspended in PBS^++^ (+ Ca^2+^ and + Mg^2+^) (137 mM NaCl, 2.7 mM KCl, 8 mM Na_2_HPO_4_, 1.8 mM KH_2_PO_4_, 0.9 mM CaCl_2_, 0.5 mM MgCl_2_). 2 × 10^5^ cells were transferred into individual reaction tubes. After a 5 min incubation on ice, cells were pelleted by centrifugation and then resuspended and incubated with PBS^++^ containing, C2IIa^488^, C2I^405^ or Ib^488^ for 30 min on ice as indicated in the individual experiment. Cells were washed again with PBS^++^ prior to flow cytometry measurements using the BD FACSCelesta™ Cell Analyzer (Becton, Dickinson and Company, Franklin Lakes, NJ, USA) with BB515 and BV421 respectively for blue (488 nm) and violet (405 nm) laser. For every labeled toxin component, a corresponding dye control was included and served as a negative control (approximating the non-reacted dye after the labeling and desalting process). Preparation of the individual dye controls is described under the Materials and Methods subsection “Toxins and reagents”. Analysis of the raw data was carried out using Flowing Software 2.5.1 (Turku Bioscience, Turku, Finland) and GraphPad Prism was used for graphical representation. GraphPad Prism was also used for curve fitting and IC_50_ calculations using nonlinear regression with [inhibitor] vs. response – Variable slope (four parameters) function.

### Enzyme-linked immunosorbent assay (ELISA)

Protein-protein interactions were analyzed using titration enzyme-linked immunosorbent assays (ELISA). MaxiSorp Nunc™ Immuno modules (Thermo Fisher Scientific, Waltham, MA, USA) were coated with 60 µl of the respective interaction partner (100 nM C2IIa, PA_63_, or Ib, dilutions of human plasma or serum samples (in PBS)) and incubated overnight at 4 °C as indicated in the individual experiment. Wells were washed twice with 200 µl PBS-T (PBS + 0.05% Tween-20) and blocked with 200 µl PBS-BSA (PBS + 1% BSA (Carl Roth, Karlsruhe, Germany)) for 60 min. After an additional washing step, the coated wells were overlayed with 60 µl of a dilution series of the sample (fibrinogen, human plasma or serum, C2I or deglycosylated fibrinogen) as indicated in the individual assays and incubated for 30 min at room temperature. Human plasma samples were pretreated and activated if indicated. Therefore, samples were incubated with 10 mU/µl thrombin (Cytiva, Marlborough, MA, USA) for 30 min at room temperature. After a centrifugation step (10 min, 18213 rcf, room temperature) the supernatant was additionally filtered (0.2 μm, Avantor, Radnor, USA) and then used for experiments. Controls were performed as indicated with the highest concentrations of the respective samples. After sample incubation, wells were washed three times with PBS-T. First antibodies or antisera were diluted in PBS-BSA, added to the wells (50 µl) as indicated in the individual experiments and incubated for 30 min at room temperature. Fibrinogen α (C-7) (1:200 in PBS-BSA, Santa Cruz Biotechnology, Dallas, TX, USA); Anthrax Protective Antigen Antibody (1:200 in PBS-BSA, Thermo Fisher Scientific, Waltham, MA, USA), C2II antiserum (1:10000 in PBS-BSA [9]), , C2IN antiserum (1:10000 in PBS-BSA [77]), and Ib antiserum (1:1000 in PBS-BSA, kindly provided by M. R. Popoff) were used. After three washing steps with PBS-T, wells were incubated with 50 µl of the respective secondary antibodies goat anti-Mouse IgG (H + L) secondary antibody HRP (1:1600 in PBS-BSA, Thermo Fisher Scientific, Waltham, MA, USA) or mouse anti-rabbit IgG-HRP (1:400 in PBS-BSA, Santa Cruz Biotechnology, Dallas, TX, USA) for 30 min at room temperature. Lastly, the wells were washed again prior to absorbance measurements using the Tecan infinite M1000Pro plate reader (Tecan Trading AG, Männedorf, Switzerland) at 650 nm with 50 µl of tetramethylbenzidin (TMB) solution (Seramun, Heidesee, Germany). Absorbance values were plotted against the concentration of the overlay titration or coating dilution and a [Agonist] vs. response – Variable slope (four parameters) non-linear regression (curve fit) was applied with GraphPad Prism for graphical representation and calculation of EC_50_. *K*_D_ values were calculated also in GraphPad Prism using the method described in Eble JA [54]. In graphical representations horizontally dashed lines indicate the upper and lower limits of the applied fit while vertically dashed lines indicate either *K*_D_ or EC_50_ values as indicated.

### Surface plasmon resonance

Surface plasmon resonance (SPR) was used to analyze the binding of fibrinogen to C2IIa using the Reichert SPR7500DC SPR spectrometer (Reichert Technologies. Buffalo, NY, USA). A SPR sensor chip CMD500m (XanTec bioanalytics GmbH, Düsseldorf, Germany) was conditioned as instructed by the manufacturer prior immobilization. During experiments a flow rate of 25 µl/min was used with PBS + 0.005% Tween-20 as running buffer. C2IIa was immobilized via amine-coupling. It was diluted to a concentration of 40 µg/ml in 5 mM sodium acetate pH 4.7 and injected for 6 min over one flow cell after activating the carboxyl groups with EDC/NHS. This yielded an immobilization level of approx. 1800 response units (RU) (and 7000 RU on a second chip, compare Supplementary Fig. 8). Of note, a slow drift of the signal was observed over time, which was explained by the heptameric nature of C2IIa, from which a small fraction might have dissociated from the chip (overnight signal decreased e.g. from 7000 RU to 6300 RU). Fibrinogen was dialyzed in running buffer. For a single cycle kinetic measurement fibrinogen was injected at the indicated concentrations for 4 min with a dissociation phase of 5 min in between the injections, followed by a final dissociation phase of 60 min. Recombinantly produced FH15-19 was used as a non-binding control (NBC). Multi cycle measurements were performed by injections of a concentration series with association phases of 5 min each followed by a dissociation phase of 45 min. The reproducibility of measurements was tested by injecting multiple cycles of 2.5 µM fibrinogen for 4 min each with a dissociation phase of 45 min. For graphical representation, SPR response curves are aligned for each sample application. Only reference subtracted sensorgrams are shown throughout.

### Binding affinity measurement in solution

Binding affinity experiments were performed to analyze the binding of labelled fibrinogen to C2IIa in solution via the MonolithX (NanoTemper, Munich, Germany). Sample preparations were performed as instructed by the manufacturer. Fibrinogen was labeled using the Protein Labeling Kit RED-NHS 2nd Generation according to the manufacturer’s instructions using PBS + 0.005% Tween-20 as assay buffer. C2IIa was used in a serial 1:2 dilution (in PBS) starting at 600 nM and ending at 0.018 nM. Labelled fibrinogen was added in a constant concentration (20 nM, diluted in PBS + 0.005% Tween-20) to reach a final assay buffer concentration of PBS + 0.0025% Tween-20. Samples were incubated for 10 min at room temperature and measured using the Monolith Premium Capillaries (NanoTemper, Munich, Germany). *K*_D_ values were calculated using the MO.Control 2.6.3 software (NanoTemper, Munich, Germany). For graphical representation measured values (ratio 670 nm/650 nm) were exported and normalized to the top and bottom limit of a [Agonist] vs. response – Variable slope (four parameters) non-linear regression (curve fit). Bottom limit of the curve fit was subtracted from the values, which were then normalized to the difference between top and bottom limit of the curve fit. The normalized values represent the unbound fibrinogen fraction, which was given as a percentage. Graph was generated with GraphPad Prism and the same fit as before applied for graphical representation.

### Collection of human blood

All experiments were performed in accordance with the Helsinki declaration (World medical association declaration of Helsinki Ethical principles for medical research involving human subjects, JAMA, 2013), under ethical approval (number 370/24) by the local ethics committee of Ulm University, and after receiving written informed consent. Venous blood was collected from healthy donors in either EDTA-, citrate-, or serum monovettes (Sarstedt, Nümbrecht, Germany).

### Rotational thromboelastometry (ROTEM) analysis

The impact of C2IIa on the hemostatic system was investigated using ROTEM (Rotem delta, Werfen, Germany). Citrated blood was exposed to C2IIa at a low (3.32 nM) and high (332 nM) concentration immediately before measurement. To focus on pure plasmatic clotting and to exclude platelet contribution, the FIBTEM setup using cytochalasin D was chosen.

In an alternative blood-free approach to investigate the direct effect of C2IIa on fibrinogen, fibrinogen (final concentration at 5 µM) was added to serum-free MEM buffer in absence or presence of C2IIa (3.32 nM) and ROTEM was started. For control, 0.2 U/ml thrombin (Merck, Darmstadt, Germany) were added to fibrinogen in serum-free MEM buffer to demonstrate feasibility of the procedure.

### EDTA-plasma and serum generation

EDTA-anticoagulated human blood was used to generate EDTA-plasma. Samples were immediately centrifuged at 2200 rcf for 15 min at 4 °C. For serum generation human blood was allowed to clot for 30 min at RT and eventually centrifuged the same way. Plasma and serum samples were aliquoted and stored at -80 °C until usage.

### Deglycosylation of fibrinogen

Remove-iT^®^ PNGase F was used for deglycosylation of fibrinogen (New England Biolabs, Ipswich, MA, USA). The reaction was performed according to the protocol of the manufacturer at 37 °C for 75 min as follows: 40 µg of fibrinogen was diluted with water in a total of 20 µl with the addition of 2 µl 10X GlycoBuffer2 and 4 µl of Remove-iT^®^ PNGase F. To avoid fibrinogen denaturation, the denaturation step at 55 °C of the protein was skipped and no DTT was added. Chitin Magnetic Beads (New England Biolabs, Ipswich, MA, USA) were also used according to the manufacturer for PNGase F removal with slight deviations: PBS was used to wash the beads and deglycosylated fibrinogen (Fib_deglyc_.) was removed from the beads once and no follow up washes were combined. As a control, fibrinogen was subjected to the same treatment regimen with the only difference, that Remove-iT^®^ PNGase F was not added during the incubation step, this was denoted as “treated” fibrinogen (Fib_treat_.). The concentration of all Fib samples (Fib_deglyc_. and Fib_treat_.) was determined after the deglycosylation, and then used as required for the individual experiments.

### Statistics

All experiments were performed with at least three independent experiments if not explicitly stated otherwise. Individual replicates (n) from the experiments were used as indicated. Non-parametric one-way ANOVA with Dunnett’s multiple comparison test was performed as indicated (GraphPad Prism, Version 10.2.2). p values were indicated as follows: not significant (ns) *p* ≥ 0.05, * *p* < 0.05, ** *p* < 0.01, *** *p* < 0.001, **** *p* < 0.0001.

## Supplementary Information

Below is the link to the electronic supplementary material.

Supplementary Material 1

## Data Availability

Any data reported in this paper is available from the corresponding author upon reasonable request.
