# Supplementary material for: A host defense role for Fibrinogen by direct binding of Clostridium botulinum C2 toxin
Source: Cell Mol Life Sci. 2026 May 19;83(1):208. doi: 10.1007/s00018-026-06253-7 (PMC13187091; doi:10.1007/s00018-026-06253-7)
Supplement: Supplementary file 1 — Supplementary Material 1 [file 18_2026_6253_MOESM1_ESM.docx]

# Supplement


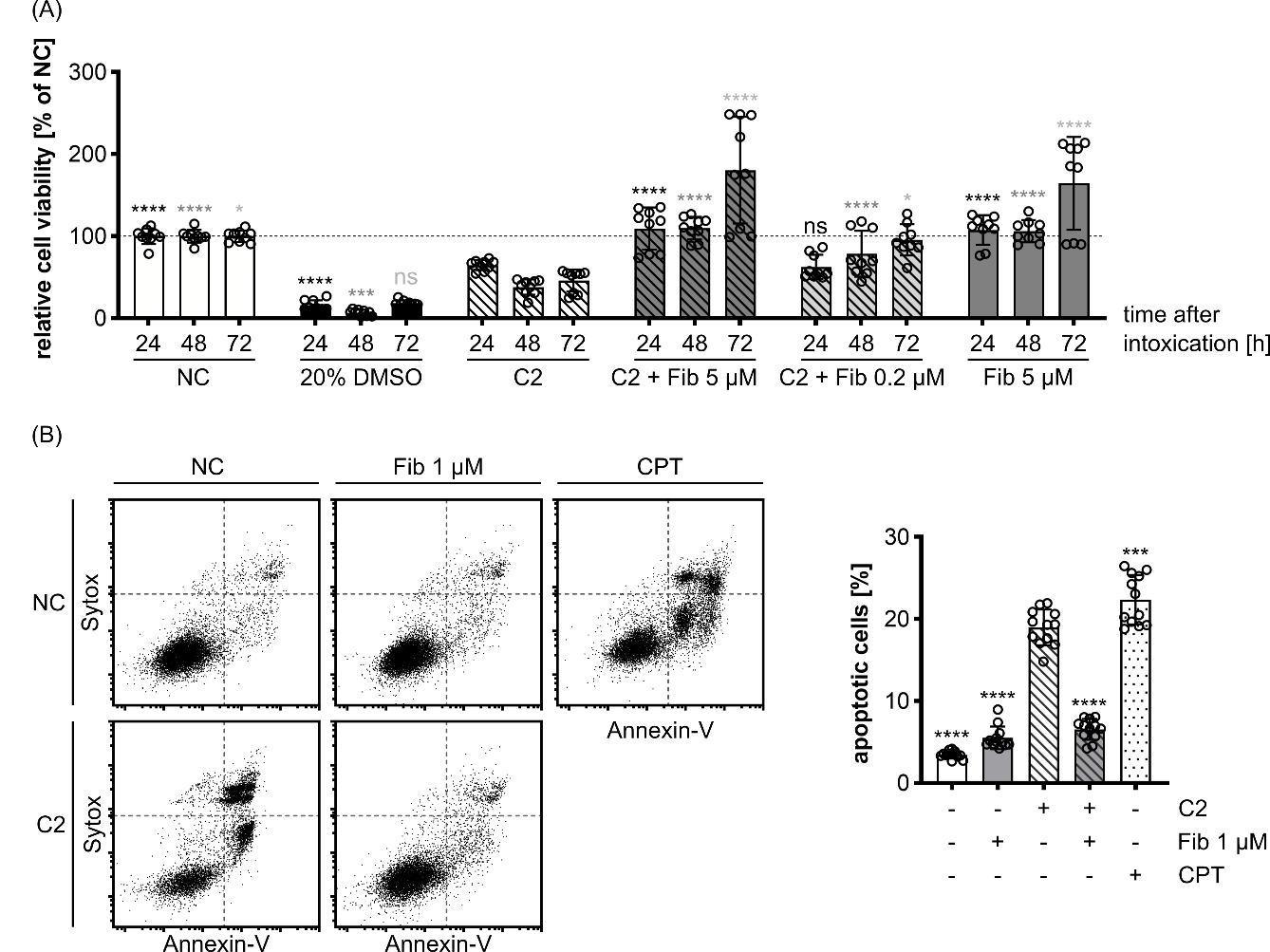


**Supplementary Figure 1**: Fib protects HeLa cells from C2 toxin-induced cell death. **(A)** Same experiment as shown in **Figure 1E**, additional conditions are included**.** Cells were treated as indicated with Fib, C2 toxin (2 nM C2I + 3.32 nM C2IIa) or DMSO (assay control) or left untreated for negative control (NC). Cell viability was measured by using the MTS assay for each condition after one, two and three days and normalized for every timepoint to the NC. Values are given as mean ± SD (*n* = 9) of triplicates from three individual experiments. Statistical analysis was always performed within the respective timepoints (depicted as differently shaded significance symbols in black, dark grey and light grey for 24 h, 48 h and 72 h respectively). **(B)** Same experiment as shown in **Figure 1F.** Representative dot plots of cells from an apoptosis assay. Cells were treated for 24 h with Fib (1 µM), C2 toxin (2 nM C2I + 3.32 nM C2IIa) or Camptothecin (CPT, 10 µM) as indicated. Cells were detached, stained and analyzed in the flow cytometer for apoptosis (Annexin-V positive, Sytox negative) or necrosis (Annexin-V positive, Sytox positive). Quantification of the percentage of apoptotic cells as in **Figure 1F**. Values are given as mean ± SD (*n* = 12) of triplicates from four individual experiments. Statistical analysis was performed compared to the corresponding group treated with C2 toxin in the absence of Fib by using non-parametric one-way ANOVA in combination with Dunnett’s correction for multiple comparison (ns *p* ≥ 0.05, * *p* < 0.05, *** *p* < 0.001, **** *p* < 0.0001).


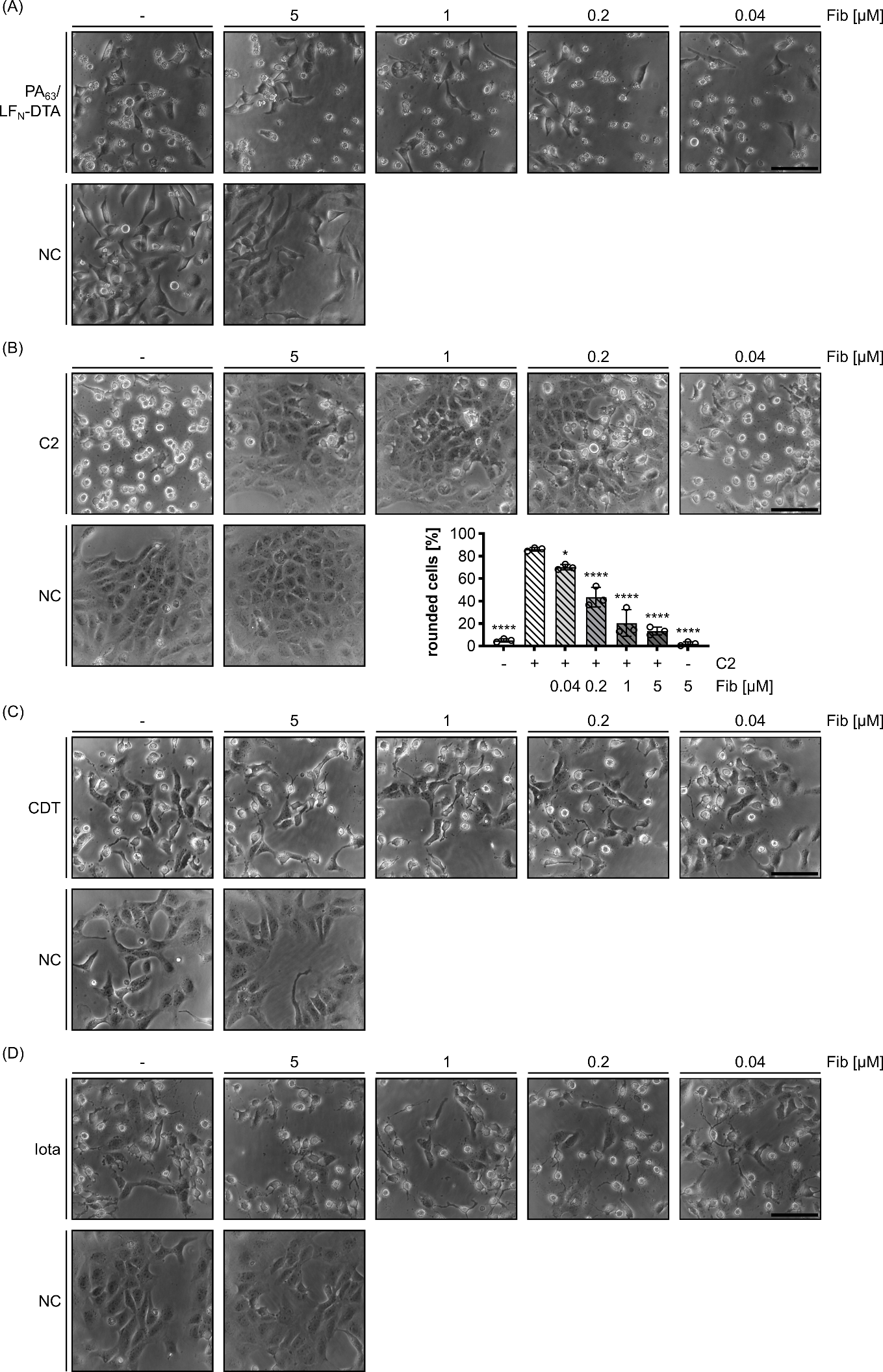


**Supplementary Figure 2**: Fib does not inhibit the related binary toxins PA_63_/LF_N_-DTA, CDT or Iota. The same experiments as shown in **Figure 3** are used. **(A)** Representative pictures of HeLa cells after 3 h incubation with PA_63_/LF_N_-DTA (0.5 nM PA_63_ + 0.25 nM LF_N_-DTA) in the absence or presence of the indicated concentration of Fib. Untreated cells served as a negative control (NC). **(B)** Representative pictures of Vero cells after 8 h incubation with C2 toxin (2 nM C2I + 3.32 nM C2IIa) in the absence or presence of the indicated concentration of Fib. Untreated cells served as a negative control (NC). Quantification of the results is depicted in the bottom right. Values are given as mean ± SD (*n* = 3) of triplicates from one representative experiment. Statistical analysis was performed compared to the C2 control by using non-parametric one-way ANOVA in combination with Dunnett’s correction for multiple comparison (* *p* < 0.05, **** *p* < 0.0001). **(C)** Representative pictures of Vero cells after 4 h incubation with CDT (2.8 nM CDTa + 3 nM CDTb) in the absence or presence of the indicated concentration of Fib. Untreated cells served as a negative control (NC). **(D)** Representative pictures of Vero cells after 3 h incubation with Iota toxin (0.7 nM Ia + 0.94 nM Ib) in the absence or presence of the indicated concentration of Fib. Untreated cells served as a negative control (NC). Scale bar corresponds to 100 µm.


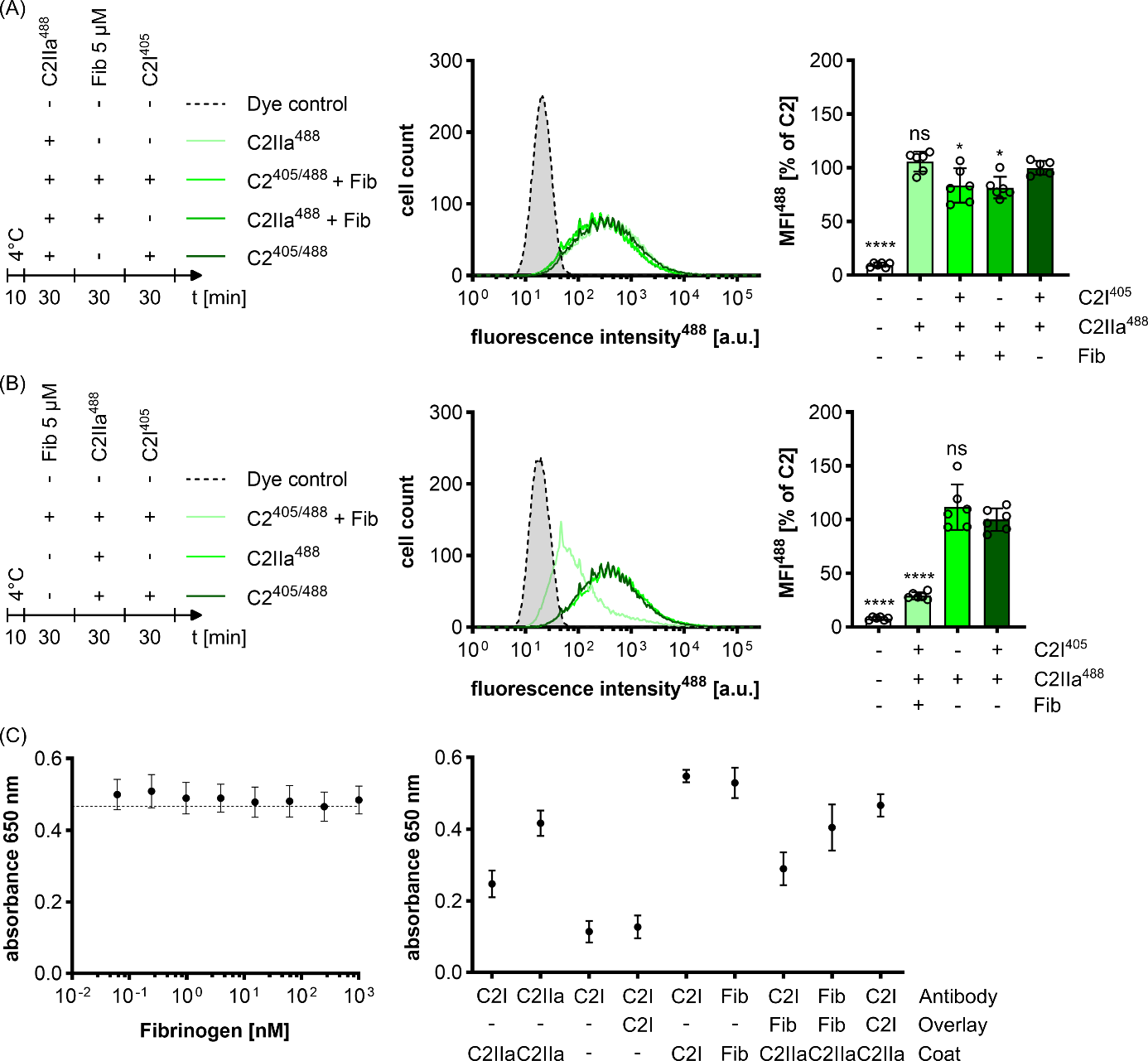


**Supplementary Figure 3**: Fib protects cells from C2IIa binding but does not influence already bound C2IIa and does not prevent C2I from binding to C2IIa. **(A)** On the left-hand side is a schematic representation of the experimental timeline. HeLa cells were detached and sequentially treated with C2IIa^488^ (13 nM), Fib (Fib 5 µM) and C2I^405^ (16 nM) as indicated. Fluorescence intensities (FI) were measured via flow cytometry. Histograms of the fluorescence intensities are depicted in the middle. The corresponding bar graph on the right-hand side shows median fluorescence intensities (MFI) normalized to C2^405/488^. **(B)** Schematic display of the experimental setup. HeLa cells were detached and sequentially treated with the same components from (a) in a different order as indicated. Fluorescence intensities (FI) were measured and depicted as in (a). Statistical analysis was performed compared to the C2 control by using non-parametric one-way ANOVA in combination with Dunnett’s correction for multiple comparison (ns *p* ≥ 0.05, * *p* < 0.05, **** *p* < 0.0001). **(C)** ELISA of coated C2IIa with Fib and C2I as a sequential overlay. C2IIa was coated to the plate, Fib was added in a sequential 1:4 dilution (1000 nM to 0.06 nM) as a first overlay followed by a second overlay with C2I (500 nM). Values are given as mean ± SD (*n* = 6) of duplicates from three individual experiments. Horizontally dashed line indicates the mean value of the interaction between C2IIa and C2I in the absence of Fib. Controls of the assay are depicted on the right-hand side. Proteins for coating and overlay and corresponding antibodies or antisera were used as indicated with the highest concentration of the respective samples. Values are given as mean ± SD (*n* = 6) of duplicates from three individual experiments.


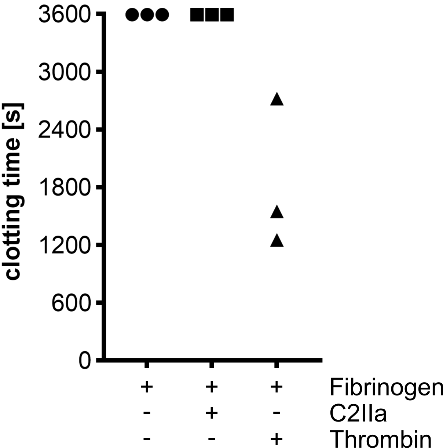


**Supplementary Figure 4**: Fib does not aggregate to fibrin without controlled proteolytic activation. ROTEM analysis of aggregation of 5 µM Fib diluted in serum-free medium with and without the addition of thrombin. The aggregation time was measured in 3 individual experiments. Of note, the measurement was stopped after 1 h (i.e. 3600 s) and no observed aggregation within the measured timeframe was depicted at this timepoint (all measurements without thrombin addition).


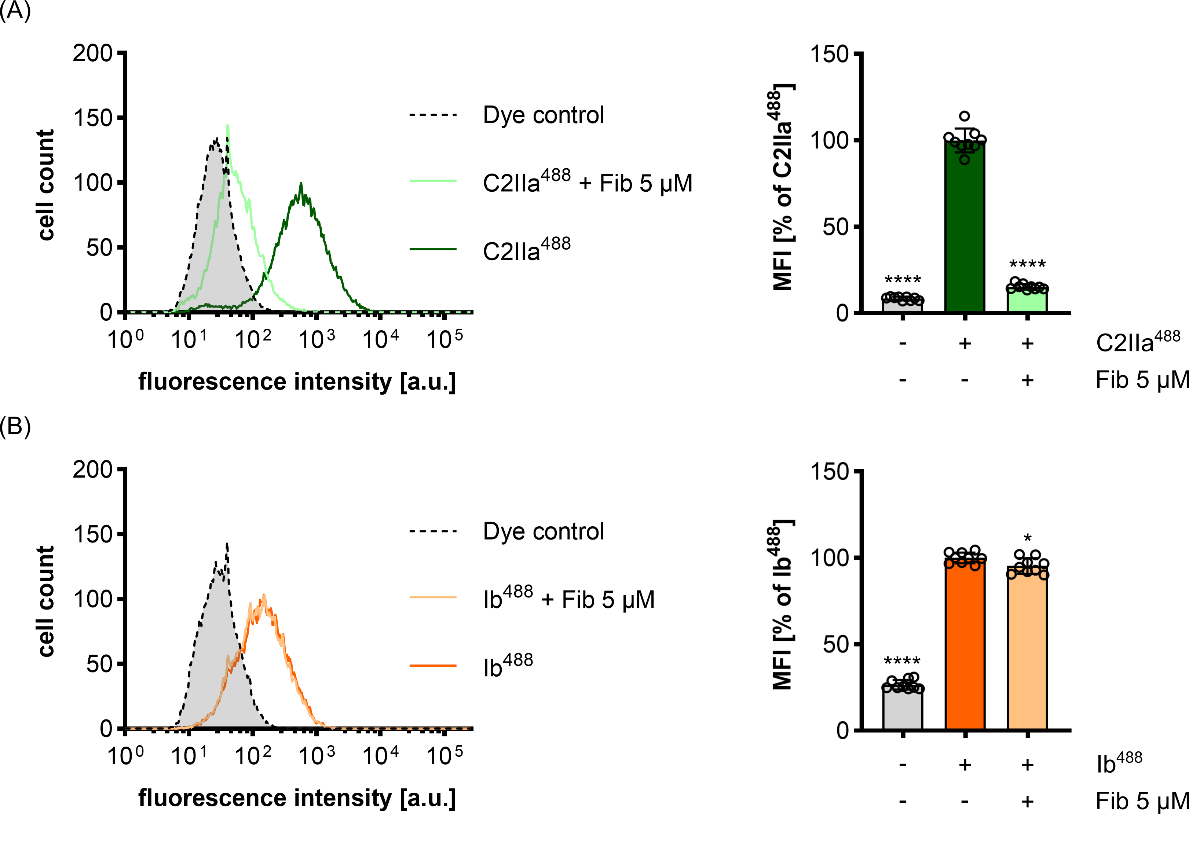


**Supplementary Figure 5**: Fib inhibits binding of C2IIa but not Ib to Vero cells **(A)** Binding of C2IIa^488^ to Vero cells. Cells were detached, cooled on ice to prevent toxin uptake and treated with C2IIa^488^ (13 nM) and Fib (5 µM) to measure the fluorescence intensities (FI) via flow cytometry. Histograms of the fluorescence intensities of are depicted on the left. The corresponding bar graph with median fluorescence intensities (MFI) normalized to C2IIa^488^ is depicted on the right. Values are given as mean ± SD (*n* = 9) of triplicates from three individual experiments. **(B)** Binding of Ib^488^ to Vero cells. Cells were treated as in (A) with Ib^488^ (7.9 nM) and Fib (5 µM). Depiction is as in (A). Statistical analysis was performed compared to the C2IIa^488^/Ib^488^ control by using non-parametric one-way ANOVA in combination with Dunnett’s correction for multiple comparison (* *p* < 0.05, **** *p* < 0.0001).


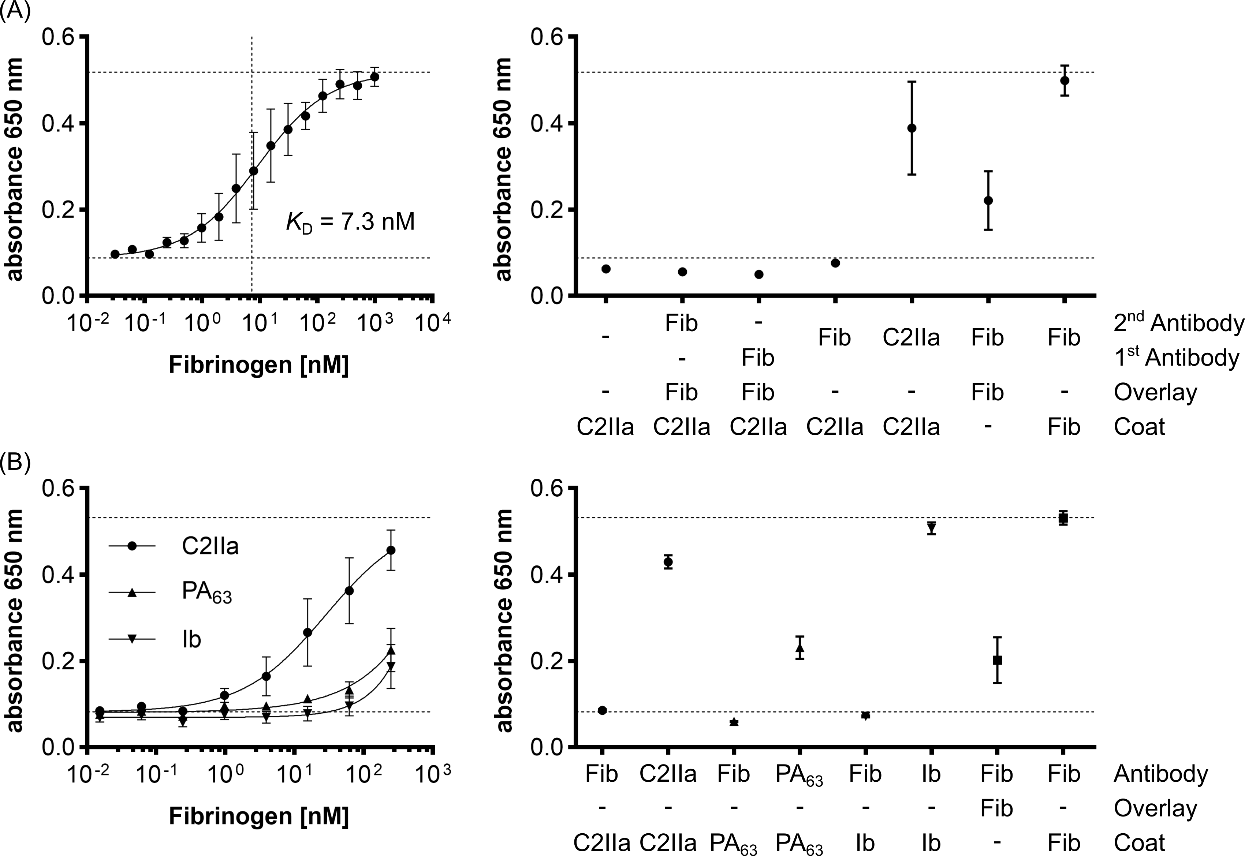


**Supplementary Figure 6**: Control graphs of ELISA experiments with Fib and C2IIa, PA_63_ or Ib. **(A)** ELISA of coated C2IIa and Fib as in **Figure 7A**. Controls from the same assay are depicted on the right-hand side. Proteins for coating, overlay and corresponding antibodies or antisera were used as indicated with the highest concentration of the respective sample. Values are given as mean ± SD (*n* = 8) of duplicates from four individual experiments. **(B)** ELISA of coated C2IIa, PA_63_ or Ib and as in **Figure 7B**. Controls from the same assay on the right-hand side. Proteins for coating and overlay and corresponding antibodies or antisera were used as indicated with the highest concentration of the respective sample. Values are given as mean ± SD (*n* = 8) of duplicates from four individual experiments.


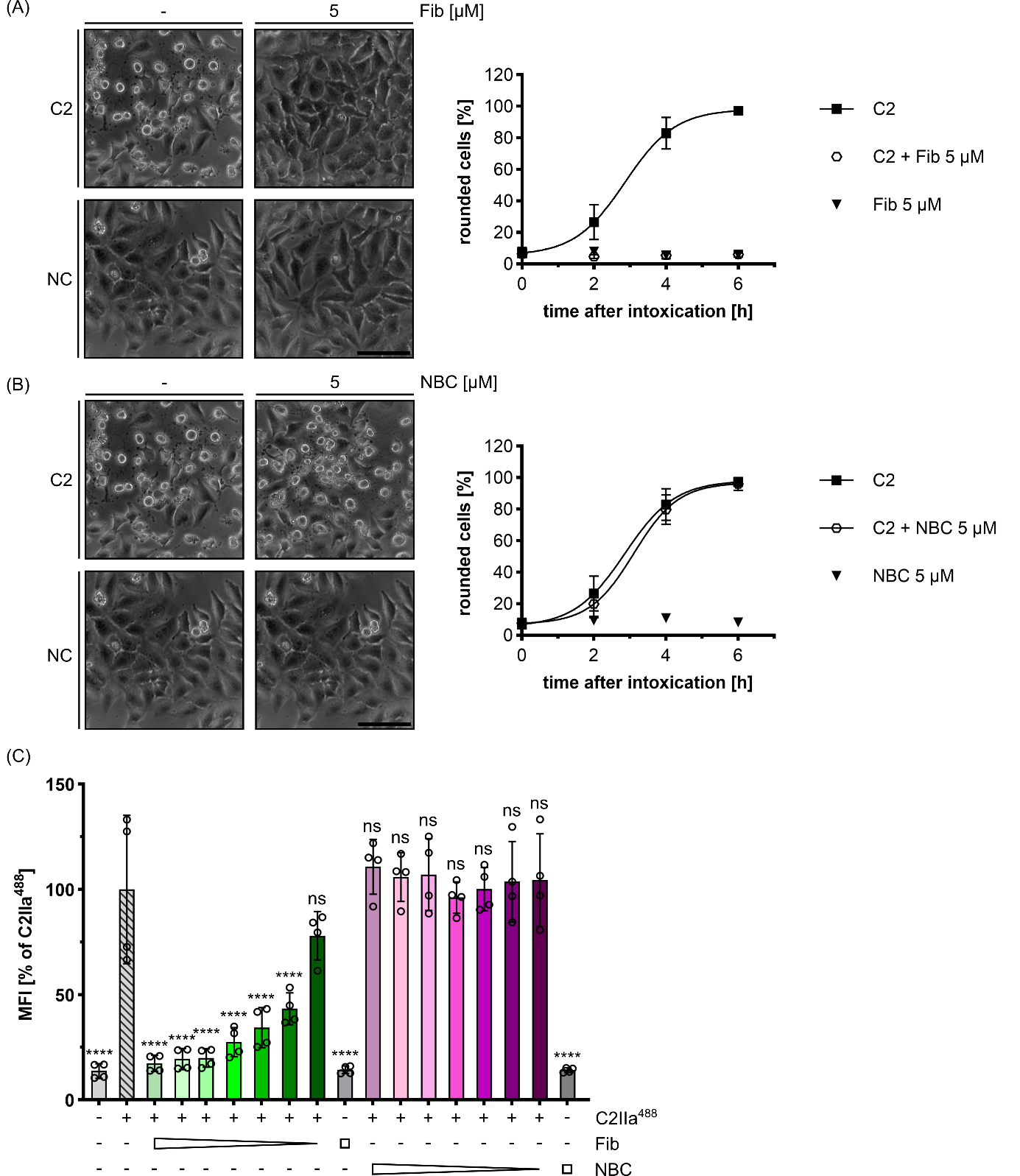


**Supplementary Figure 7**: Non-binding control (NBC) does not inhibit C2 toxin. **(A)** Representative pictures of HeLa cells after 4 h incubation treated with the indicated concentrations of Fib or C2 (2 nM C2I + 3.32 nM C2IIa) are depicted left. Untreated cells served as a negative control (NC). On the right, pictures were taken at the indicated time points and the percentage of rounded cells from the total cell number was quantified. Values are given as mean ± SD (*n* = 3) of triplicates from two individual experiments. **(B)** Binding assay of C2IIa^488^ to HeLa cells. Cells were detached and treated with C2IIa^488^ (13 nM) and Fib or NBC as indicated (5000 nM, 1000 nM, 500 nM, 100 nM, 50 nM, 25 nM and 5 nM). Bar Graph with median fluorescence intensities (MFI) normalized to C2IIa^488^ is depicted. Values are given as mean ± SD (*n* = 4) of duplicates from two individual experiments. Statistical analysis was performed compared to the C2IIa^488^ control by using non-parametric one-way ANOVA in combination with Dunnett’s correction for multiple comparison (ns *p* ≥ 0.05, **** *p* < 0.0001).


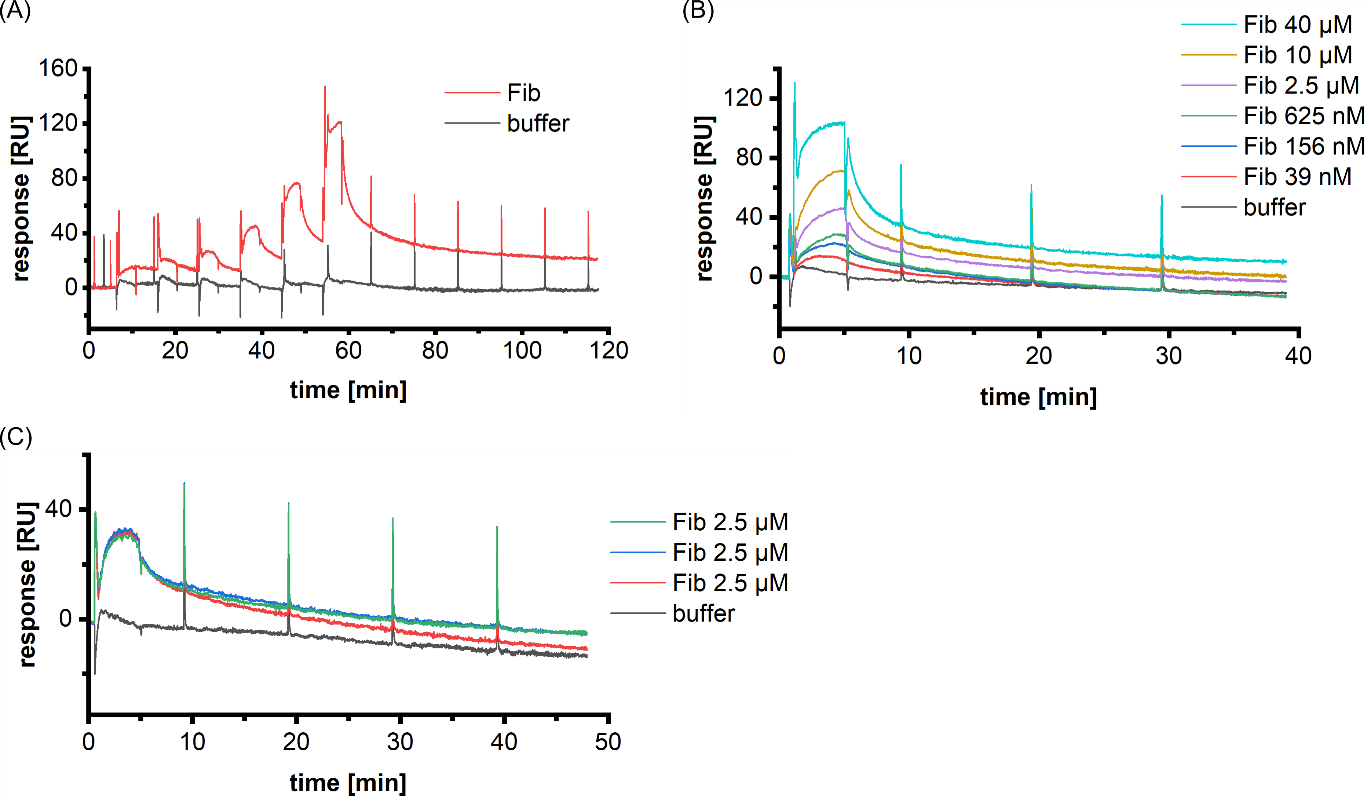


**Supplementary Figure 8**: SPR measurements on chip B. 7000 RU of C2IIa were immobilized and Fib was injected at indicated concentrations diluted in running buffer (buffer). **(A)** Single cycle kinetics with 4 min injections of increasing concentrations of Fib (39 nM; 156 nM; 625 nM; 2.5 µM; 10 µM and 40 µM) followed each by a 5 min dissociation phase and a final dissociation phase of 60 min. One buffer measurement is included as control in the same graph. **(B)** Multi cycle measurement with injections of different concentrations of Fib followed by a dissociation phase with running buffer for at least 45 min. Regeneration conditions were avoided due to the heptameric nature of C2IIa, as stated earlier. **(C)** Three consecutive injections of the same concentration of Fib (2.5 µM) for 4 min each followed by a dissociation phase with a flow of running buffer for 45 min each.


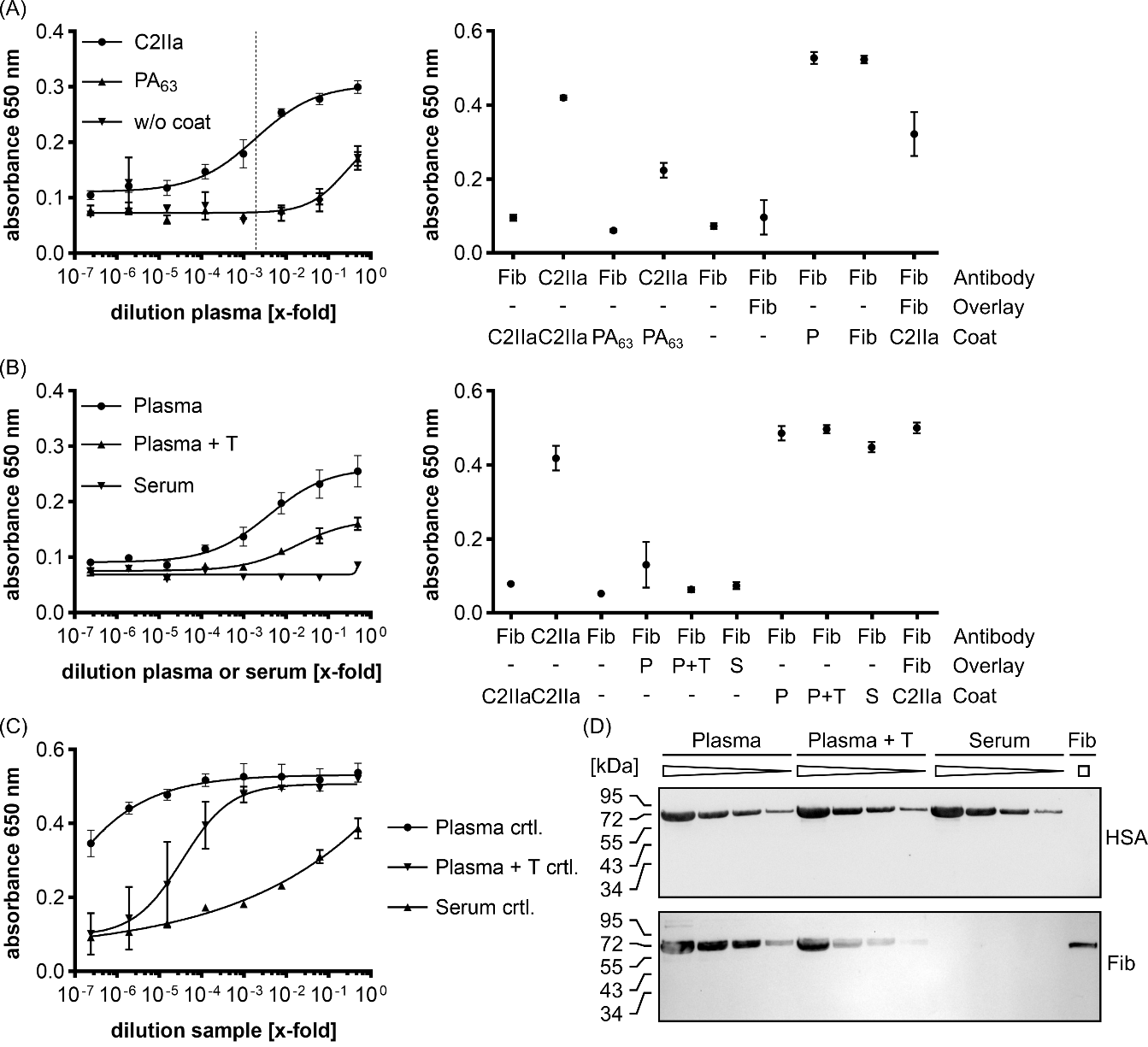


**Supplementary Figure 9**: Fib in human plasma interacts with C2IIa. **(A)** ELISA of coated C2IIa and PA_63_ with human plasma as depicted in **Figure 8A**. Controls from the same assay are depicted on the right hand side. Proteins for coating, overlay and corresponding antibodies or antisera were used as indicated with the highest concentration of the respective sample. Values are given as mean ± SD (*n* = 6) of duplicates from three individual experiments. **(B)** ELISA of coated C2IIa and human plasma, thrombin (T) activated plasma or serum as depicted **in Figure 8B**. Controls from the same assay are depicted on the right hand side. Proteins for coating, overlay and corresponding antibodies or antisera were used as indicated with the highest concentration of the respective sample. Values are given as mean ± SD (*n* = 6) of duplicates from three individual experiments. **(C)** ELISA of coated human plasma, thrombin activated plasma or human serum in sequential 1:8 dilution starting from a 1:2 dilution. Fib levels of the coated samples were detected with the respective antibody. Values are given as mean ± SD (*n* = 3 or 4) from two individual experiments. **(D)** Representative Western blot of Fib levels in human plasma, thrombin activated plasma or human serum. Samples were loaded as indicated in a descending dilution series (0.2 µl, 0.1 µl, 0.05 µl, 0.02 µl). Fib (500 ng) served as a control. Human serum albumin (HSA) was detected to show equal loading.

#
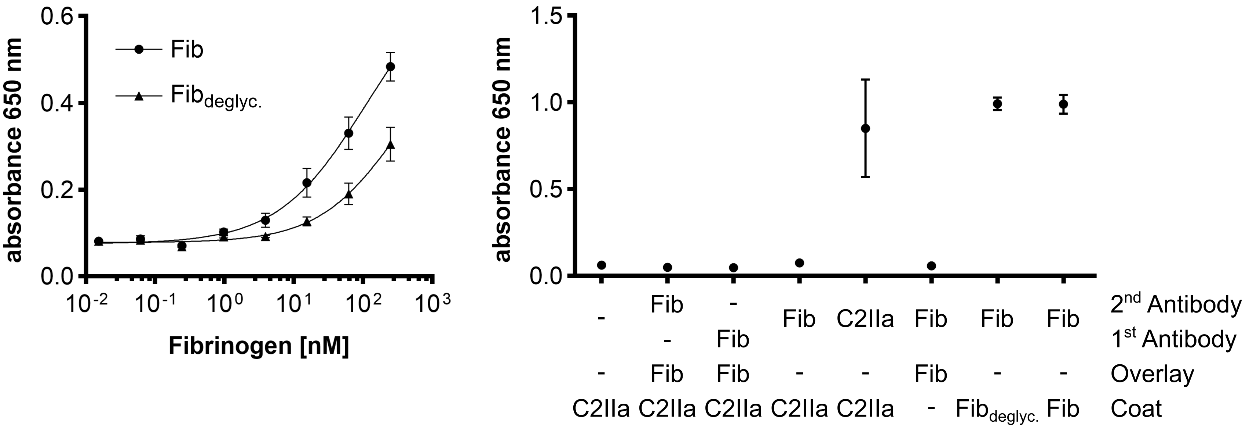


**Supplementary Figure 10**: N-glycosylation of Fib mediates the affinity of Fib to C2IIa. ELISA of coated C2IIa with Fib and Fib_deglyc_. as depicted in **Figure 9B**. Controls from the same assay are depicted on the right hand side. Proteins for coating, overlay and corresponding antibodies or antisera were used as indicated with the highest concentration of the respective sample. Values are given as mean ± SD (*n* = 6) of duplicates from three individual experiments.
